# Supplementary material for: Dating Alphaproteobacteria evolution with eukaryotic fossils
Source: Nat Commun. 2021 Jun 3;12:3324. doi: 10.1038/s41467-021-23645-4 (PMC8175736; doi:10.1038/s41467-021-23645-4)
Supplement: Supplementary file 1 — Supplementary Information [file 41467_2021_23645_MOESM1_ESM.pdf]

Supplementary data for

Dating *Alphaproteobacteria* evolution with eukaryotic fossils

Sishuo Wang[1], Haiwei Luo[1]

[1]Simon F. S. Li Marine Science Laboratory, School of Life Sciences and State Key Laboratory of Agrobiotechnology, The Chinese University of Hong Kong, Shatin, Hong Kong SAR

This file includes the following items

Supplementary Tables 1 to 3
Supplementary Notes 1 to 3
Supplementary Figures 1 to 16
Supplementary references

Table of contents

Supplementary Tables 1 to 3
Supplementary Table 1……..…………………………………………………… 3
Supplementary Table 2 ……………………………………………………… 7
Supplementary Table 3 ……………………………………………………… 8
Supplementary Notes 1 to 3
Supplementary Note 1: Supplementary Methods
1.1 Genome selection ………..………………..…………………………… 9
1.2 Molecular dating ……………..……..………………………………... 10
1.3 Reconstruction of ancestral hosts ……………..……..…....……………… 13
1.4 Data visualization ………………..……………… 14
Supplementary Note 2: Calibration information
2.1 Calibrations for the mitochondria-based strategy ....……..………… 15
2.2 Calibrations for the cyanobacteria-based strategy ....……..………… 19
Supplementary Note 3: Additional discussion
3.1 Limitations of the approach adopted in Shih et al., 2017 ….....……..…...…… 22
3.2 Limitations of calibrating the evolution of bacteria based on the divergence times of their modern hosts ………………………………………………….…….. 22
3.3 Implications for the evolution of *Rickettsiales* …………………..……..…..… 23
3.4 Selection of the clock model ……………………….………………..………..… 24
Supplementary Figures …………………………………..…………..………..… 26
Supplementary references

Supplementary Table 1. Genome sources.

Taxonomy	Species	Nuclear genome	Mitogenome
Metazoa	Homo sapiens	Ensembl release 96 [2]	
Metazoa	Gallus gallus	Ensembl release 96 [2]	
Metazoa	Branchiostoma floridae	UniProt [3]	
Metazoa	Amphimedon queenslandica	Ensembl Metazoa release 46 [2]	
Fungi	Candida albicans	UniProt [3]	
Fungi	Ustilago maydis	Ensembl Fungi release 46 [2]	
Fungi	Pleurotus ostreatus	UniProt [3]	
Fungi	Spizellomyces punctatus	UniProt [3]	
Amoebozoa	Acanthamoeba castellanii	Ensembl Protists release 43 [2]	
Amoebozoa	Dictyostelium discoideum	Ensembl Protists release 43 [2]	
Amoebozoa	Polysphondylium pallidum	dictyBase [4]	
Archaeplastida	Arabidopsis thaliana	PLAZA 4.0 [5]	MitoCOGs
Archaeplastida	Oryza sativa	PLAZA 4.0 [5]	MitoCOGs
Archaeplastida	Physcomitrella patens	PLAZA 4.0 [5]	MitoCOGs
Archaeplastida	Ostreococcus tauri	PLAZA 2.0 [6]	MitoCOGs
Archaeplastida	Chondrus crispus	Ensembl Plants release 46 [2]	MitoCOGs
Archaeplastida	Porphyra umbilicalis	UniProt [3]	[7]
Archaeplastida	Cyanidioschyzon merolae	Ensembl Plants release 43 [2]	MitoCOGs
Archaeplastida	Cyanophora paradoxa	http://cyanophora.rutgers.edu/cyanophora/	MitoCOGs
Heterokontophyta	Phytophthora infestans	Ensembl Protists release 46 [2]	MitoCOGs
Heterokontophyta	Thalassiosira pseudonana	Ensembl Protists release 46 [2]	
Discoba	Andalucia godoyi	https://megasun.bch.umontreal.ca/Andalucia_godoyi/ [8]	MitoCOGs
Discoba	Histiona aroides		MitoCOGs
Discoba	Jakoba bahamiensis		MitoCOGs
Discoba	Jakoba libera		MitoCOGs
Discoba	Reclinomonas americana		MitoCOGs
Discoba	Seculamonas ecuadoriensis		MitoCOGs
Malawimonadida	Malawimonas jakobiformis		MitoCOGs
SAR	Symbiodinium minutum	https://marinegenomics.oist.jp/ v1.2 [9]	
SAR	Paramecium tetraurelia	Ensembl Protists release 45 [2]	
SAR	Oxytricha trifallax	OxyDB (http://oxy.ciliate.org/)	
SAR	Reticulomyxa filosa	Ensembl Protists release 43 [2]	
SAR	Elphidium margaritaceum	MMETSP (transcriptomes) [10]	
α-proteobacteria	Acidiphilium angustum ATCC 35903	GCA_000701585.1	
α-proteobacteria	alpha proteobacterium AAP81b	GCA_001295935.1	
α-proteobacteria	alpha proteobacterium Q-1	GCA_000710935.1	
α-proteobacteria	alpha proteobacterium RS24	GCA_000469155.1	
α-proteobacteria	alphaproteobacterium SCGC AAA288-N07	GCA_000513055.1	
α-proteobacteria	alphaproteobacterium sp. HIMB5	GCA_000299095.1	

α-proteobacteria	Anaplasma phagocytophilum HZ	GCA_000013125.1
α-proteobacteria	Asticcacaulis excentricus CB 48	GCA_000175215.2
α-proteobacteria	Bartonella quintana RM-11	GCA_000294715.1
α-proteobacteria	Caedibacter 37-49	GCA_001898725.1
α-proteobacteria	Caedibacter 38-128	GCA_001898705.1
α-proteobacteria	Caedibacter varicaedens	GCA_001192655.1
α-proteobacteria	Candidatus Arcanobacter lacustris	GCA_000970895.1
α-proteobacteria	Candidatus Caedibacter acanthamoebae	GCA_000743035.1
α-proteobacteria	Candidatus Finniella lucida	GCA_004210305.1
α-proteobacteria	Candidatus Jidaibacter acanthamoeba	GCA_000815465.1
α-proteobacteria	Candidatus Midichloria mitochondrii IricVA	GCA_000219355.1
α-proteobacteria	Candidatus Nucleicultrix amoebiphila FS5	GCA_002117145.1
α-proteobacteria	Candidatus Odyssella thessalonicensis L13	GCA_000190415.2
α-proteobacteria	Candidatus Paracaedibacter acanthamoebae isolate PRA3	GCA_000742835.1
α-proteobacteria	Candidatus Paracaedibacter symbiosus	GCA_000757605.1
α-proteobacteria	Candidatus Pelagibacter IMCC9063	GCA_000195085.1
α-proteobacteria	Candidatus Pelagibacter ubique HTCC8051	GCA_000472605.1
α-proteobacteria	Citromicrobium JLT1363	GCA_000186705.2
α-proteobacteria	Ehrlichia canis Jake	GCA_000012565.1
α-proteobacteria	Elioraea tepidiphila DSM 17972	GCA_000378465.1
α-proteobacteria	endosymbiont of Peranema	GCA_004210275.1
α-proteobacteria	endosymbiont of Stachyamoeba	GCA_003932735.1
α-proteobacteria	Granulibacter bethesdensis CGDNIH1	GCA_000014285.2
α-proteobacteria	Hirschia baltica ATCC 49814	GCA_000023785.1
α-proteobacteria	Hyphomicrobium denitrificans ATCC 51888	GCA_000143145.1
α-proteobacteria	Inquilinus limosus DSM 16000	GCA_000423185.1
α-proteobacteria	Jannaschia EhC01	GCA_001650845.1
α-proteobacteria	Ketogulonicigenium vulgare WSH-001	GCA_000223375.1
α-proteobacteria	Kordiimonas gwangyangensis DSM 19435 - JCM 12864	GCA_000375545.1
α-proteobacteria	Meganema perideroedes DSM 15528	GCA_000374145.1
α-proteobacteria	Methylobacterium extorquens AM1	GCA_000022685.1
α-proteobacteria	Methylocystis SC2	GCA_000304315.1
α-proteobacteria	Micavibrio aeruginosavorus ARL-13	GCA_000226315.1

α-proteobacteria	Neorickettsia sennetsu Miyayama	GCA_000013165.1
α-proteobacteria	Oceanibaculum indicum P24	GCA_000299935.1
α-proteobacteria	Oceanicaulis HTCC2633	GCA_000152745.1
α-proteobacteria	Orientia tsutsugamushi Boryong	GCA_000063545.1
α-proteobacteria	Paracoccus denitrificans PD1222	GCA_000203895.1
α-proteobacteria	Parvibaculum lavamentivorans DS-1	GCA_000017565.1
α-proteobacteria	Parvularcula bermudensis HTCC2503	GCA_000152825.2
α-proteobacteria	Pelagibacter sp. HIMB058	GCA_000012345.1
α-proteobacteria	Pelagibacterium halotolerans B2	GCA_000230555.1
α-proteobacteria	Phenylobacterium zucineum HLK1	GCA_000017265.1
α-proteobacteria	Rhizobium leguminosarum bv. trifolii WSM1689	GCA_000517605.1
α-proteobacteria	Rhodobacter sphaeroides 2.4.1	GCA_000012905.2
α-proteobacteria	Rhodopseudomonas palustris TIE-1	GCA_000020445.1
α-proteobacteria	Rhodospirillum centenum SW	GCA_000016185.1
α-proteobacteria	Rhodospirillum rubrum ATCC 11170	GCA_000013085.1
α-proteobacteria	Rhodovibrio salinarum DSM 9154	GCA_000515255.1
α-proteobacteria	Rickettsia typhi Wilmington	GCA_000008045.1
α-proteobacteria	Rubritepida flocculans DSM 14296	GCA_000425365.1
α-proteobacteria	Ruegeria ANG-R	GCA_000813985.1
α-proteobacteria	Sneathiella glossodoripedis JCM 23214	GCA_000616095.1
α-proteobacteria	Sphingomonas wittichii	GCA_000016765.1
α-proteobacteria	Thalassobaculum salexigens DSM 19539	GCA_000423805.1
α-proteobacteria	Thalassospira profundimaris WP0211	GCA_000300275.1
α-proteobacteria	Wolbachia endosymbiont of Culex quinquefasciatus Pel	GCA_000073005.1
α-proteobacteria	Wolbachia endosymbiont of Onchocerca ochengi	GCA_000306885.1
Magnetococcales	Magnetococcus marinus MC-1	GCA_000014865.1
Magnetococcales	Magnetofaba australis IT-1	GCA_002109495.1
β-proteobacteria	Burkholderia thailandensis E264	GCA_000012365.1
β-proteobacteria	Nitrosospira multiformis ATCC 25196	GCA_000196355.1
γ-proteobacteria	Alteromonas lipolytica	GCA_001758465.1
γ-proteobacteria	Spongiibacter tropicus DSM 19543	GCA_000420325.1
cyanobacteria	Crinalium epipsammum PCC 9333	GCA_000317495.1
cyanobacteria	Gloeobacter kilaueensis JS1	GCA_000484535.1
cyanobacteria	Gloeobacter violaceus PCC 7421	GCA_000011385.1
cyanobacteria	Lyngbya aestuarii BL J	GCA_000478195.2
cyanobacteria	Moorea producens 3L	GCA_000211815.1
cyanobacteria	Nostoc sp. PCC 7107	GCA_000316625.1
cyanobacteria	Oscillatoria sp. PCC 10802	GCA_000332335.1

cyanobacteria	Prochlorococcus marinus CCMP1986	GCA_000011465.1
cyanobacteria	Raphidiopsis brookii D9	GCA_000175855.1
cyanobacteria	Richelia intracellularis HH01	GCA_000350105.1
cyanobacteria	Synechococcus elongatus PCC 6301	GCA_000010065.1
cyanobacteria	Thermosynechococcus elongatus BP-1	GCA_000011345.1
cyanobacteria	Xenococcus sp. PCC 7305	GCA_000332055.1
Melainabacteria	Ca. Caenarcanum bioreactoricola UASB 169	GCA_001899385.1
Melainabacteria	Ca. Melainabacteria bacterium SSGW 16SSGW 16	GCA_004769085.1
Melainabacteria	Vampirovibrio chlorellavorus Vc AZ 2	GCA_003149345.1
Sericytochromatia	Ca. Sericytochromatia bacterium GL2-5 LSPB 72	GCA_002083815.1
Sericytochromatia	Ca. Sericytochromatia bacterium S15B-MN24 RAAC 196	GCA_002083785.1

Supplementary Table 2. Comparison of the prior and posterior time estimates (mean and 95% HPD intervals) in MCMCTree analysis (the best-practiced scheme). The time estimates are given in the unit of million years.

Mito-encoded

	Prior			Posterior		
**Clade**	**Mean**	**Min**	**Max**	**Mean**	**Min**	**Max**
Mitochondria	2323.55	1606.26	2965.19	1545.26	1376.97	1718.2
Alphaproteobacteria	2435.66	1722.42	3020.49	2010.77	1752.52	2266.85
Caulobacterales	1636.71	604.607	2425.74	1163.89	1002.04	1340.86
Holosporales	1922.56	1182.07	2706.55	1359.66	1167.63	1557.48
Rhizobiales	1908.3	1211.45	2558.67	1374.5	1189.24	1573.38
Rhodobacterales	1710.37	862.324	2465.62	1154.52	983.296	1322.88
Rhodospirillales	2279.31	1608.81	2947.94	1683.98	1455.76	1901.48
Rickettsiales	2244.76	1494.87	2874.06	1741.45	1514.17	1974.93
Pelagibacterales	1838.28	950.441	2622.96	992.802	831.075	1156.88
Sphingomonadales	1881.66	1008.13	2709.25	1479.54	1265.93	1683.45

Nuclear-encoded

	Prior			Posterior		
**Clade**	**Mean**	**Min**	**Max**	**Mean**	**Min**	**Max**
Mitochondria	2095.66	1332.72	2738.23	1569.23	1447.04	1690.84
Alphaproteobacteria	2149.49	1338.16	2791.61	1832.97	1668.14	2001.16
Caulobacterales	1403.58	513.169	1992.05	1155.95	1026.44	1289.42
Holosporales	1688.45	1047.66	2348.89	1281.66	1138.33	1428.85
Rhizobiales	1642.19	990.694	2251.96	1393.42	1247.97	1532.76
Rhodobacterales	1457.52	576.239	2040.22	1028.74	906.882	1154.19
Rhodospirillales	1990.67	1316.52	2594.08	1563.41	1415.09	1721.27
Rickettsiales	1956.04	1280.77	2597.45	1607.23	1447.19	1766.86
Pelagibacterales	1557.87	609.984	2233.39	801.623	677.47	928.384
Sphingomonadales	1625.63	736.92	2334.58	1432.38	1278.68	1579.9

Supplementary Table 3. Topology tests of different topologies of the *Alphaproteobacteria* tree. These topologies correspond to those displayed in Supplementary Fig. 1C, including the best-practiced tree (tree 1) and 11 alternative topologies.

Tree	logL	deltaL	bp-RELL	p-KH	p-SH	c-ELW	p-AU
**1**	-73965.2	0	0.629 (+)	0.652 (+)	1 (+)	0.622 (+)	0.719 (+)
**2**	-73992.9	27.67	0.0035 (-)	0.0207 (-)	0.162 (+)	0.00407 (-)	0.0336 (-)
**3**	-73994.4	29.147	0.0055	0.0206 (-)	0.137 (+)	0.00566 (-)	0.0215 (-)
**4**	-73967.3	2.0707	0.35 (+)	0.348 (+)	0.87 (+)	0.356 (+)	0.444 (+)
**5**	-73995.2	30.004	0.0008 (-)	0.0148 (-)	0.128 (+)	0.0012 (-)	0.00992 (-)
**6**	-73997.2	31.956	0.0018 (-)	0.0156 (-)	0.107 (+)	0.00194 (-)	0.0078 (-)
**7**	-74005.4	40.124	0.009 (-)	0.0204 (-)	0.0605 (+)	0.00886 (-)	0.0106 (-)
**8**	-74040.5	75.26	0 (-)	0.0007 (-)	0.001 (-)	1.48e-09 (-)	0.00107 (-)
**9**	-74036.1	70.862	0 (-)	0.0017 (-)	0.0019 (-)	9.68e-06 (-)	0.00252 (-)
**10**	-74008.2	42.938	0 (-)	0.0075 (-)	0.03 (-)	0.000102 (-)	0.000837 (-)
**11**	-74040.7	75.509	0 (-)	0.0002 (-)	0.0004 (-)	4.97e-09 (-)	0.00077 (-)
**12**	-74041.1	75.908	0 (-)	0.0003 (-)	0.0004 (-)	2.27e-07 (-)	0.00192 (-)

deltaL: log likelihood difference from the maximal log likelihood in the set.
bp-RELL: bootstrap proportion using RELL method.
p-KH: *P*-value of one sided Kishino-Hasegawa test.
p-SH: *P*-value of Shimodaira-Hasegawa test.
c-ELW: Expected Likelihood Weight.
p-AU: *P*-value of approximately unbiased test.
Those marked by a sign “-” indicates that the tree is rejected by the corresponding test.
Those marked by a sign “+” indicate that the tree is not rejected by the corresponding test.

Supplementary Note 1: Supplementary Methods

1.1 Genome selection

Selection of genomes for phylogenomic reconstruction

We selected 80 genomes to determine the phylogenetic relationship of *Alphaproteobacteria* and mitochondria, including 64 alphaproteobacterial species, ten eukaryotes, and six outgroup genomes (Supplementary Table 2). We chose 64 alphaproteobacterial genomes from a recent phylogenomic study of *Alphaproteobacteria* [11] based on our following principles: i) Four fast-evolving genomes (*Candidatus* Hepatobacter penaei, *Holospora obtusa*, *Holospora undulata* and alphaproteobacterium HIMB59) and two genomes with unstable phylogenetic positions (*Tistrella mobilis* and *Geminicoccus roseus*) were removed; ii) All of the 12 and 9 genomes from *Rickettsiales* and *Holosporales*, respectively, were kept; iii) 43 genomes covering all major clades were selected to represent the diversity of *Alphaproteobacteria*. We chose ten mitochondrial genomes that are gene rich and that display relatively short branch lengths to minimize the impacts of long branch attraction, as used in a prior study [12]. These eukaryotic species were *Physcomitrella patens*, *Ostreococcus tauri, Andalucia godoyi*, *Jakoba libera*, *Jakoba bahamiensis*, *Histiona aroides*, *Seculamonas ecuadoriensis*, *Reclinomonas americana*, *Malawimonas jakobiformis*, and *Phytophthora infestans*. Six genomes from *Magnetococcales*, *Betaproteobacteria* and *Gammaproteobacteria* were used as the outgroup [11].

Eukaryotic lineages used in dating

It is important to note that, as described above, we followed Martijn *et al.*, 2018 to construct the phylogeny of *Alphaproteobacteria* and mitochondria with ten eukaryotes whose mitogenomes are gene-rich and slowly-evolving thus ideal for phylogenetic analysis. However, these ten eukaryotes, which were mainly comprised by jakobids, a group of free-living heterotrophic protists from the eukaryotic supergroup Discoba, provided little fossil information. Hence, to take the advantages of eukaryotic fossils, we included more eukaryotic taxa in the mitochondria subtree for dating. We based the topology of the mitochondria subtree in dating analysis on the general consensus understanding of the eukaryotic phylogeny (Supplementary Fig. 2A), rooted at the branch separating Amorphea (including Amoebozoa, Fungi and Metazoa) and others [13,14]. Using alternative topologies of the mitochondria subtree showed similar time estimates for *Alphaproteobacteria* (Supplementary Fig. 10).

Eukaryotic lineages were selected based on prior studies [12,15,16] to cover both important lineages and those with high-quality fossil records. We compiled two datasets: the mito-encoded dataset, which was based on the 24 genes conserved between *Alphaproteobacteria* and mitochondrial genomes, and the nuclear-encoded dataset, which was based on 22 nuclear genes transferred from the mitochondrial genomes in evolution [17] (Supplementary Data 1). Orthologs were retrieved from the MitoCOGs database and identified by BLAST search using sequences identified by Wang and Wu, 2015 as queries for the mito- and nuclear-encoded datasets, respectively, and only hits with the lowest e-values were retained (Supplementary Table 1).

The genomes in the mito-encoded dataset included the ten aforementioned genomes used in phylogenomic reconstruction, and another six genomes from Archaeplastida

(hereafter referred to as plants for simplicity) to take advantages of plant fossils (Supplementary Fig. 2A), including two flowering plants (*Arabidopsis thaliana* and *Oryza sativa*), three red algae (*Chondrus crispus*, *Porphyra umbilicalis* and *Cyanidioschyzon merolae*), and a glaucophyte (*Cyanophora paradoxa*) (Supplementary Fig. 2A). These genomes were chosen for the mito-encoded dataset because their mitogenomes were generally gene-rich and slowly-evolving [12].

As shown in Supplementary Fig. 2A, the nuclear-encoded dataset included all genomes used in the mito-encoded dataset except for the six without nuclear genomes, and additionally three amoebae, four animals, four fungi, and six from the Stramenopiles-Alveolata-Rhizaria (SAR) supergroup. Specifically, the three Amoebozoa lineages were two dictyostelids (social amoebae; *Dictyostelium discoideum* and *Polysphondylium pallidum*), and one Discosea species (flattened amoebae that move as a whole; *Acanthamoeba castellanii*). The four animals included two amniotes (*Homo sapiens* and *Gallus gallus*), a primitive chordate (*Branchiostoma floridae* [amphioxus]), and one from the early-split metazoan lineage sponge (*Amphimedon queenslandica*). The four fungi were three Dikarya species (*Candida albicans*, *Ustilago maydis* and *Pleurotus ostreatus*) and one chytrid, an early-split fungal lineage (*Spizellomyces punctatus*). The six SAR lineages consisted of a diatom (silicified microalgae; *Thalassiosira pseudonana*), two rhizarians (a diverse group of protists defined by phylogenetic evidence; *Reticulomyxa filosa* and *Elphidium margaritaceum*), two ciliates [protists characterized by the presence of cilia; *Paramecium tetraurelia* from Oligohymenophorea and *Oxytricha trifallax* from Spirotrichea [18]], and a dinoflagellate (most members are characterized by two dissimilar flagella; *Symbiodinium minutum*). Including these eukaryotes allowed using six additional fossils and directly comparing the divergence times of host-associated bacteria and their eukaryotic hosts on the dated tree (Supplementary Fig. 2A).

1.2 Molecular dating

1.2.1 MCMCTree analysis with the mitochondria-based strategy

Previous studies showed that different partitioning schemes could lead to differences in the estimated ages and precision [19,20]. Genes within the same partition are allowed to have their own pattern of among-lineage rate heterogeneity in dating analyses. We considered a single partition (where all sequences were concatenated into one partition), the full partitioning (where each gene was regarded as an independent partition), and several other partitioning strategies (where genes with similar functions or features were clustered into the same partition). Different partitioning strategies showed generally consistent posterior estimates, in particular for the nuclear-encoded dataset (Supplementary Fig. 6). In line with previous studies [15,19], the fully partitioned strategy displayed the highest precision (Supplementary Fig. 6), and was therefore used in the main analysis.

To determine the best-fit clock models, we employed the mcmc3r package [21], which uses a stepping-stone method to calculate the marginal likelihood of three different clock models implemented in MCMCTree: the strict clock model (STR), the autocorrelated model (AR; more specifically, the geometric Brownian motion rate model), and the independent rate (IR) model. This method works for the exact likelihood method, which is only available for nucleotide sequences in MCMCTree. To overcome the challenge imposed by the huge

computational burden, we followed McGowen *et al.*, 2020: i) We reduced the subsets of taxa by randomly selecting 20 and 40 species for both the mito- and nuclear-encoded datasets; ii) For each subset, we randomly selected three, five and ten genes; iii) the root prior was fixed, and no other calibrations were used (here we did not intend to calculate divergence times but simply select the best-fit model). The lowest marginal likelihoods were always obtained with the strict clock model. The autocorrelated clock model turned out to have the highest marginal likelihood in most analyses (Supplementary Fig. 13), and thus was used as the preferred model.

Phylogenetic studies have shown high rate heterogeneity among the mitochondria, *Rickettsiales* and the remaining lineages in *Alphaproteobacteria* [12,17,23] (see also Supplementary Fig. 1). We therefore set the parameter specifying the gamma distribution of the standard deviation of log-transformed rate on branches, σ, to be "1 10 1", which indicates a standard deviation of log-transformed rate on branches to be 0.1. This setting should accommodate considerable among-lineage rate variation [24]. We also set σ to be "1 1 1" (meaning a standard deviation of log-transformed rate branches of 1.0) to accommodate larger among-lineage rate heterogeneity. The results were highly consistent (*sigma* in Supplementary Fig. 5).

LG [25] and GTR [26] were used as the substitution models for amino acid and nucleotide sequences, respectively. Four discrete categories of gamma distribution were applied to account for rate heterogeneity across sites. The mean substitution rate for each partition was based on the average substitution rates of all partitions calculated by codeml (or baseml for nucleotides) to inform the Dirichlet-gamma prior ("rgene_gamma").

1.2.2 MCMCTree analysis with additional alphaproteobacterial lineages represented by metagenome-assembled genomes (MAGs)

We further performed a molecular dating analysis of *Alphaproteobacteria* with a wider taxonomic sampling. This was done by using 16 MAGs (MarineAlpha3-12 and MarineProteo1) according to the study [12], many of which are early-split alphaproteobacterial lineages, in addition to the genomes used in the above analysis. We did not use MarineAlpha1 and MarineAlpha2 as they are likely closely related to "core alphaproteobacterial lineages" [12]. We employed the same procedures as described above for phylogenomic reconstruction and MCMCTree analysis (Supplementary Fig. 8).

1.2.3 MCMCTree analysis with the cyanobacteria-based strategy

Thirteen oxygenic cyanobacteria genomes were selected based on a recent study [27] (Supplementary Fig. 2B; Supplementary Table 1) after discarding three and six relatively closely related species from the *Pleurocapsales* and *Nostocales* respectively. We also included five non-photosynthetic cyanobacteria lineages [28] as the outgroup of oxygenic cyanobacteria (Supplementary Fig. 2B; Supplementary Table 1). Twenty-five conserved genes between *Alphaproteobacteria* and cyanobacteria used by others [27,29] were analyzed. To keep consistency with the mitochondria-based analysis, the same settings in MCMCTree analysis were kept for the cyanobacteria-based method. Three calibration points within cyanobacteria were collected and used in the cyanobacteria-based dating analysis of *Alphaproteobacteria* (see Supplementary Note 2.2). The best-practiced calibration set (the

dating scheme *Sánchez-Baracaldo 2017* in Supplementary Data 2) was chosen based on careful review of the fossil evidence used in previous studies (see Supplementary Note 2.2). Note that the study Sánchez-Baracaldo et al., 2017 might inappropriately assign the maximum constraints and phylogenetic positions for certain cyanobacteria calibrations, which were not adopted in our analysis (see Supplementary Note 2.2).

1.2.4 Composite chronograms

The mean age of each node in the composite (joint) chronograms was calculated by integrating all the posterior ages obtained from different competing dating schemes (*Phan*, *Max-1*, *Max-2*, *Single partition*, *IR*, and best-practiced) using MCMCTree. The joint 95% HPD intervals were calculated using the R package BayesTwin [30]. By integrating the estimates from different analyses, the composite chronograms can help better appreciate the uncertainties associated with Bayesian relaxed molecular clock analysis, as used in other studies [15,19].

1.2.5 Infinite-sites plots

To assess whether the posterior density of the divergence times estimated by MCMCTree had reached the limiting distribution when the data had infinite sites, we employed the strategy employed by the previous studies [31,32]. This theory predicts that given infinite amount of sequence data, the posterior mean of divergence times and the posterior credibility interval width should form a linear relationship, the slope in the infinite-sites plot denoting uncertainties in the estimated posterior ages that could not be reduced by sequence data alone. Hence, if in an infinite-sites plot the scatter points approach a straight line, the maximum amount of information has been obtained from the molecular data. This indicates that further reduction of the uncertainty in the posterior age estimates can be only achieved by using more informative calibration priors [31].

1.2.6 Molecular dating using PhyloBayes

Molecular clock analysis of the divergence time of *Alphaproteobacteria* was also performed using PhyloBayes v4.1b [33] with both the log-normal autocorrelated ("-ln") rates and uncorrelated rates gamma multipliers ("-ugam") clock models, respectively. To keep consistency with the settings in the MCMCTree analysis, the LG model was used as the substitution model with four discrete gamma categories, and a soft tail of 2.5% to each of the upper and lower calibration bound was applied to all calibration points. Two independent chains were run, each with 100,000 iterations. The first 10,000 iterations were discarded as burn-in, and the rest was sampled every 50 iterations. Convergence was assessed using the "tracecomp" function implemented in PhyloBayes. The maximum bipartition discrepancies (maxdiff) across the two chains in all analyses were smaller than 0.1, indicating good runs [34].

1.2.7 Molecular dating using BEAST

Moreover, we co-estimated the divergence times and phylogeny of *Alphaproteobacteria* lineages using BEAST v2.6.3 [35] based on the same amino acid sequences used for MCMCTree analysis. The tree inferred from our phylogenomic analysis (dayhoff4 recoded; see Supplementary Fig. 1A) was used as the input tree. We used a discrete Gamma model

with four categories to account for rate heterogeneity among sites for each individual gene, BLOUSUM62 as the substitution model, and a relaxed log normal clock as the clock model. The prior distribution for each model parameter was inferred from a birth-death process. Note that unlike MCMCTree and PhyloBayes, BEAST does not allow soft time bounds, thus the posterior ages are allowed to change only within the given maximum and minimum bounds. Two independent MCMC chains were run, each with 100,000,000 generations sampling every 1,000 generations. The first 25% generations were discarded as burn-in. All other parameters were set as default.

1.2.8 Different topologies of the *Alphaproteobacteria* phylogeny

It is important to take into account the uncertainties associated with tree topology in divergence time estimation. Although our detailed phylogenomic reconstruction supports the topology shown in Fig. 1, there are several important unknowns about the phylogeny of the *Alphaproteobacteria*. The three main lineages that often displayed distinct phylogenetic positions in previous studies are mitochondria, *Holosporales* and *Pelagibacterales* [36]. For these three lineages, we considered two, two and three different topologies, respectively, according to previous studies (Supplementary Fig. 1C). The mitochondrial lineage was suggested to branch outside the *Alphaproteobacteria* [12] (Topologies 1-3, 7-9), but are placed as a sister group of *Rickettsiales* according to traditional views [17,37,38] (Topologies 4-6, 10-12). The *Holosporales* was originally thought to be closely related to *Rickettsiales* [17,39] (Topologies 7-12), but were shown to be closely related to *Rhodospirillales* when amino acid composition biases were corrected [11,23] (Topologies 1-6). The *Pelagibacterales* was firstly identified as the sister group of mitochondria [40] (Topologies 3, 6, 9, 12). However, later studies showed that their phylogenetic affiliation likely resulted from compositional biases, which, if accounted for, would result in a pattern where *Pelagibacterales* was within a clade with *Sphingomonadales*, *Rhizobiales*, *Caulobacterales* and *Rhodobacterales* (Topologies 2, 5, 8, 11) [16,41]. Consistent with recent studies with a more complete taxon sampling [11,23], our phylogenomic analysis placed *Pelagibacterales* after its split with *Sphingomonadales* but prior to the divergence of *Rhizobiales*, *Caulobacterales* and *Rhodobacterales* (Topologies 1, 4, 7, 10).

To compare different tree topologies, we first built the phylogenetic trees constrained by the above 12 topologies using IQ-Tree ("-g"), based on which we performed topology tests using five different methods implemented in IQ-Tree: bootstrap proportion (BP) [42], Kishino-Hasegawa (KH) test [43], Shimodaira-Hasegawa (SH) test [44], expected likelihood weights (ELW) [45], and the approximately unbiased (AU) test [46]. The KH, SH and AU tests give *P*-values, and accordingly we can reject a tree if its *P*-value < 0.05. The other two methods, bp-RELL and c-ELW, return posterior weights instead of *P*-values.

1.3 Ancestral lifestyle reconstruction of the *Rickettsiales*

A total of 2,321 16S rRNA gene sequences classified to *Rickettsiales* were downloaded from NCBI Genbank database (last accessed: June, 2020). These sequences were grouped into operational taxonomic units (OTUs) based on an identity cutoff of 97% (we also repeated the analysis with a more stringent cutoff of 98.7% [Supplementary Fig. 14]) [47]. One sequence was randomly selected as the representative for each OTU. Ancestral hosts were inferred

using the MCMC method from the multistate module in BayesTraits v3.0.2 [48] based on the isolation hosts of extant members of *Rickettsiales*. The analysis was run for 1,100,000 iterations with the first 100,000 runs discarded as the burn-in. We calculated the marginal likelihoods with the stepping-stone sampler implemented in BayesTraits with 100 stones each sampled for 1,000 iterations. The statistical significance of transition rates was assessed based on the log-transformed Bayes Factor (logBF) calculated as twice the difference of the log-transformed marginal likelihood between the two tested models [48].

1.4 Data visualization

Visualization of phylogenetic trees was performed using iTOL v4 [49], FigTree v1.4.3 (http://tree.bio.ed.ac.uk/software/figtree), MEGA v7 [50], TreeGraph v2.5 [51], and the R package ggtree v2.0.4 [52]. Multiple sequence alignments were visualized using BioEdit v7.0.5.3 [53]. The calibration density was visualized with MCMCTreeR [54].

Supplementary Note 2: Calibration information

2.1 Calibration points for the mitochondria-based strategy

Note that for all calibration points (Supplementary Fig. 2A) in MCMCTree analysis, both the minimum and maximum bounds are soft and there is a probability of 2.5% that the age is beyond the bound in our settings. The only exception is the dating scheme *Max-2* where only a minimum bound is available for some calibration points (Supplementary Data 2). In this case, a Cauchy distribution is set by default in MCMCTree, denoted as $L(t_L, p, c, p_L)$ where t_L indicates the minimum bound, p determines how far the mode of the distribution goes from the minimum, c determines how sharply the distribution decays to zero, and p_L indicates the probability that the minimum bound is violated. We used $p = 0.1$, $c = 0.1$ and $p_L = 0.01$ as default, which means that both the mode and median of the distribution are at $(1 + p)t_L = 1.1t_L$.

Node: Root (*Alphaproteobacteria*/mitochondria and *Magnetococcales*)
Datasets: mito- and nuclear-encoded
Minimum age: 1000 Ma
Maximum age: 3000 Ma
Justification: The soft minimum of the root, which corresponds to the split of *Alphaproteobacteria*/mitochondria and *Magnetococcales*, was based on the fossil of *Bangiomorpha pubescens*, so far the oldest fossil that can be confidently assigned to a major eukaryotic clade (red algae) [55]. We constrained the minimum age based on the date of a shale layer in the Artic Bay formation, which is 1092 ± 59 Mya [56], thus roughly 1000 Ma. The soft maximum age was set as 3000 Ma. Because prior studies estimated the age of the *Alphaproteobacteria* from at earlies 2400 Ma, we think that 3000 Ma should be large enough to account for the uncertainties in the root age.
Alternatives: We also used three different alternative maxima for the root age, thus three calibrations 3500-1000 Ma, 4000-1000 Ma, and 4500-1000 Ma. Their results converged to the time estimates with the aforementioned root prior of 3000-1000 Ma (Supplementary Fig. 5). This suggests that 3000 Ma as a maximum constraint age on the root is sufficiently large to account for the uncertainties in the root age.

Node: Crown group of Angiospermae (flowering plants) (Node 1 in Supplementary Fig. 2A; both mito- and nuclear-encoded datasets)
Locality and Stratigraphy level: Cowleaze Chine Member, Isle of White
Minimum Age: 125 Ma
Maximum Age: 250 Ma
Justification: Tricopolate pollen is the most ancient evidence of angiosperms. Following Clark et al., 2011, we set the pollen to the Cowleaze Chine Member of the Vectis Formation, corresponding to a minimum time of 126.3 ± 0.4 Ma. The soft maximum time constraint was derived from sediments devoid of angiosperm-like pollen below their first report in the Middle Triassic, corresponding to 247.1 Ma ± 0.2 Ma [57].

Node: Crown group of Embryophyta (land plants) (Node 2 in Supplementary Fig. 2A; both mito- and nuclear-encoded datasets)

Locality and Stratigraphy level: Qusaiba-1 core from the Quasim formation of northern Saudi Arabia
Minimum Age: 450 Ma
Maximum Age: 509 Ma
Justification: Trilete spores are the oldest evidence of embryophytes known to date. We followed Clarke *et al.*, 2011 and Morris et al., 2018 dated these to 450 Ma, representing the minimum age of the crown group of embryophytes. The soft maximum constraint was placed at the Bright Angel Shale of the Tonto Group of Arizona with an age of 507.2-509 Ma [60], thus, the upper limit to 509 Ma [61].

Node: Total group of Florideophyceae (Node 3 in Supplementary Fig. 2A; both mito- and nuclear-encoded datasets)
Locality and Stratigraphy level: Doushantuo Formation, southern China
Minimum Age: 550 Ma
Maximum Age: 1891 Ma
Justification: The anatomically preserved florideophyte fossils found at Doushantuo Formation displayed features that resemble reproductive structures of modern corallines [62]. Stratigraphically constrained by Nantuo glaciation and Ediacaran fossils, the age of Doushantuo fossils were estimated to be between 600 and 550 Ma [63]. We followed Parfrey *et al.*, 2011, and used 550 Ma as the minimum age of the total group of Florideophyceae. As to the soft maximum, we followed Betts *et al.*, 2018 and Morris et al., 2018 to set it as 1891 Ma, which is based on the earliest fossil record of simple eukaryotes [65–67], when, to our knowledge, no evidence of anything as complex as multicellular algae has been reported. The maximum age of the formation is based on the rocks it overlies, dated at 1823 Ma ± 68 Ma [68].
Alternatives: The soft maximum was removed in the dating scheme *Max-2* (Supplementary Data 2).

Node: Crown group of Rhodophyta (red algae) (Node 4 in Supplementary Fig. 2A; both mito- and nuclear-encoded datasets)
Locality and Stratigraphy level: Lower Hunting Formation, Somerset Island, arctic Canada
Minimum Age: 1033 Ma
Maximum Age: 1891 Ma
Justification: The fossils of *Bangiomorpha pubescens* represent the oldest fossil that can be confidently assigned to a major eukaryotic clade. Based on the simple multicellular fossils with reproductive cell and differentiated holdfasts, people described *Bangiomorpha pubescens* as a Bangiales red algae [55], and used it as the calibration point of the red algae crown group in other studies [64,69]. We constrained the minimum age based on the date of a shale layer in the Artic Bay formation, which is 1092 ± 59 Mya [56]. Following Betts *et al.*, 2018 and Morris et al., 2018, we set the maximum as 1891 Ma (see the node of the total group of Florideophyceae).
Alternatives: Some argued that the features considered to be characteristic of *Bangiomorpha* might also be found in other red algae [15]. Thus, we followed Betts *et al.*,

2018 to assign the minimum age of the total group (Node a in Supplementary Fig. 2A), instead of the crown group, of red algae to be 1033 Ma (scheme *Max-1* in Supplementary Data 2). We also removed the soft maximum in the dating scheme *Max-2* (Supplementary Data 2).

Node: Crown group of Foraminifera (Node 5 in Supplementary Fig. 2A; nuclear-encoded dataset)
Locality and Stratigraphy level: The Chapel Island Formation, Newfoundland, Canada
Minimum age: 525 Ma
Maximum age: 1891 Ma
Justification: The fossils of *Platysolenites cooperi* are considered the oldest one in Foraminifera. The wall composition indicates that *P. cooperi* is an agglutinating foraminifera [70]. *P. cooperi* was discovered in the latest Ediacaran to Lower Cambrian in Newfoundland, the Chapel Island formation. According to the latest geological timescale [71], a minimum constraint of 525.5 Ma was set to this formation, thus the crown group of the Foraminifera. Following Betts *et al.*, 2018 and Morris et al., 2018, we set the maximum as 1891 Ma (see the node of the total group of Florideophyceae).

Node: Crown group of Amniota (mammals, birds, and reptiles) (Node 6 in Supplementary Fig. 2A; nuclear-encoded dataset)
Locality and Stratigraphy level: Joggins Formation of Nova Scotia, Canada
Minimum age: 312 Ma
Maximum age: 332 Ma
Justification: The oldest fossils that are assigned to Amniota are those of *Hylonomus lyelli* Dawson. Its minimum age was set to be 318 Ma based on the date of the Joggins Formation. We followed the study [72], and assigned the maximum age to be 332.9 Ma, the same as the fossiliferous Little Cliff Shale of the East Kirkton locality, where no fossils of reptilians are detected.
Alternatives: Despite little controversy in the maximum age of this node, to be conservative and to keep consistency with other nodes within the Metazoa, we removed the maximum age of this node in the dating scheme *Max-1* (Supplementary Data 2).

Node: Crown group of Chordata (Node 7 in Supplementary Fig. 2A; nuclear-encoded dataset)
Locality and Stratigraphy level: Haikou [Yuanshan Fm (formerly Qiongzhusi)], China
Minimum Age: 520 Ma
Maximum Age: 636 Ma
Justification: We followed Benton *et al.*, 2015 to set the minimum and maximum time constraints of the Chordata crown group. The minimum date was based on the fossils of *Haikouichthys ercaicunensis* [73], which were found in the Chengjiang Biota within the Yu'anshan Member of the Heilinpu Formation [74]. There are many Lagerstätten (sedimentary deposits displaying extraordinary fossils with exceptional preservation) in the Lantian Biota preserving the biota, but none of these fossils shows any characteristics that could assign them to the Chordata crown group or even the eumetazoan total group [72]. Thus, the

appearance time of Chordata should not be earlier than the maximum age of the Lantian Biota, which is 636 Ma [75].

Alternatives: As some argued for a more ancient origin of Chordata [76], we removed the maximum age for this node in the dating scheme *Max-1* (Supplementary Data 2).

Node: Crown group of Metazoa (animals) (Node 8 in Supplementary Fig. 2A; nuclear-encoded dataset)

Locality and Stratigraphy level: White Sea Formation, Russia

Minimum Age: 550 Ma

Maximum Age: 833 Ma

Justification: The fossil of *Kimberella quadrata*, which belongs to Bilateria, is considered as the oldest metazoan's fossil. A lower limit of 550 Ma was established for *Kimberella quadrata* [77], and thus was used as the minimum age of Metazoa. A maximum age was established from the Svanbergfjellet Formation of Spitsbergen [78] and the Bitter Springs Formation of central Australia [79] which preserve a variety of fossils assigned to eukaryotes (e.g., multicellular algae, sphaeromorph acritarchs) but show no evidence of total group metazoans. The absolute age of the Bitter Springs Formation was dated at 827 ± 6 Mya, therefore we used 833 Ma as the maximum constraint of Metazoa, as used in the previous studies [15,19]. Some studies based the minimum time of the animal crown group on the fossil lipids 24-isopropylcholestane and 26-methylstigmastane, dating back to 630 Mya. The reason was that these biomarkers were thought to be only associated with sponges [64], the presumably earliest-branching animal lineage [80,81]. However, these biomarkers were later found to be common in rhizaria, an early-branching lineage of eukaryotes, hence the affinities of these biomarkers are ambiguous [82].

Alternatives: According to some molecular dating studies [83,84], animals might have originated 300-500 Ma earlier than the above estimate. Therefore, we alternatively removed the maximum constraint for this node (scheme *Max-1* in Supplementary Data 2).

Node: Total group of Fungi (or crown group of Opisthokonta; Node 9 in Supplementary Fig. 2A; nuclear-encoded dataset)

Locality and Stratigraphy level: Brock Inlierm, the Northwest Territories, Canada

Minimum age: 890 Ma

Maximum age: 1891 Ma

Justification: The oldest fungal fossil came from the shale of Grassy Bay Formation (Shaler Supergroup, Arctic Canada), described as *Ourasphaira giraldae*. It dates to 1010–890 Mya. *O. giraldae* shows septate hyphae that are characteristic of the Dikarya but that are also found in a few lineages of the Zoopagomycota, Mucoromycota, Chytridiomycota and Blastocladiomycota [85]. Distinct from Dikarya, in the microfossil specimens of *O. giraldae*, researchers did not detect septa that are regularly distributed along the hyphae [85]. These suggest that *O. giraldae* either is in a clade of the total group of fungi or belongs to the stem group of Dikarya [85]. The oldest report of a fossil with features typical to fungi is found in the Ongeluk Formation, which dates back to 2400 Mya [86]. However, this was questioned by Berbee *et al.*, 2017 because the morphology and deep marine habitat of the fossils were inconsistent with predictions from phylogenetic analysis. Further, its biological activities

were thought to be suspicious as it lacks organic components [87]. To be conservative, we assigned a minimum time constraint of 890 Mya to the total group of fungi, i.e., the crown group of Opisthokonta. Following Betts *et al.*, 2018 and Morris et al., 2018, we set the maximum as 1891 Ma (see the node of the total group of Florideophyceae).
Alternatives: The soft maximum was removed in the dating scheme *Max-2* (Supplementary Data 2).

Node: Crown group of Dikarya (Ascomycota and Basidiomycota) (Node 10 in Supplementary Fig. 2A; nuclear-encoded dataset)
Locality and Stratigraphy level: Rhynie, Aberdeenshire, Scotland, Lower Devonian
Minimum age: 400 Ma
Maximum age: 1891 Ma
Justification: The earliest uncontroversial fossil that belongs to Dikarya is the fossil of *Paleopyrenomycites devonicus,* which shows key characteristics related to Ascomycota. The estimated date of the fossil was based on that of the Rhynie Chert system, which is ~400 Ma [88,89]. Following Betts *et al.*, 2018 and Morris et al., 2018, we set the maximum as 1891 Ma (see the node of the total group of Florideophyceae).
Alternatives: The soft maximum was removed in the dating scheme *Max-2* (Supplementary Data 2).

2.2 Calibration points for the cyanobacteria-based strategy

Note that there are four calibration points used in the cyanobacteria-based strategy (Supplementary Fig. 2B). Only one of them (i.e., the root) has both minimum and maximum time bounds available. For the remaining three internal calibration points, only a minimum time bound is available, and by default, in MCMCTree, the minimum bound is represented using a truncated Cauchy distribution, denoted as $L(t_L, p, c, p_L)$. Here, t_L indicates the minimum bound, p determines how far the mode of the distribution goes from the minimum, c determines how sharply the distribution decays to zero, and p_L indicates the probability that the minimum bound is violated. We used $p = 0.1$, $c = 0.1$ and $p_L = 0.01$ as default, which means that both the mode and median of the distribution are at $(1 + p)t_L = 1.1t_L$.

Node: *Pleurocapsales* total group (Node 1 in Supplementary Fig. 2B)
Minimum age: 1700 Ma
Maximum age: -
Justification: For the minimum age, it was based on the estimated age of the microfossils of *Pleurocapsales* found in Hebei, China, which dates back to ca. 1700 Mya [90], as used in some studies [91,92]. Note that these studies also applied a maximum constraint based on fossils of coccoid and filamentous cells, which, however, ignores the gap in time between the first appearance of an apomorphic character and its first fossilization [93]. This could lead to a false impression of precision. Hence, following Zhang et al., 2021, we left open the maximum bound of this calibration point.
Alternatives: This calibration point is not included in the dating schemes *Wolfe 2019* and *Wolfe 2019 (GOE)* (Supplementary Data 2) simply because these two schemes are according

to Wolfe and Fournier, 2018 which did not use this calibration point.

Node: *Nostocales* total group (Node 2 in Supplementary Fig. 2B)
Minimum age: 1600 Ma
Maximum age: -
Justification: Akinetes are considered to be characteristic of the *Nostocales* [96]. We assigned the minimum age of this calibration point based on the fossils described as akinetes preserved in McArthur Group of Northern Australia [97] dating back to ~1600 Ma. We followed Zhang et al., 2021 to leave the maximum age open for the same reason described for the node of the *Pleurocapsales* total group above.
Alternatives: We also tried two different minimum time constraints, 2100 Ma based on the fossils preserved in the Franceville Group of Gabon [98], and 1200 Ma based on the Middle Proterozoic Dismal Lakes Groups, Arctic Canada [99]. These two estimates are used in Sánchez-Baracaldo et al., 2014 [the dating schemes *Sánchez-Baracaldo 2014* and *Sánchez-Baracaldo 2014 (GOE)*] and Wolfe and Fournier, 2018 [the dating schemes *Wolfe 2018* and *Wolfe 2018 (GOE)*], respectively.

Node: Oxygenic cyanobacteria total group (Node 3 in Supplementary Fig. 2B)
Minimum age: 3000 Ma
Maximum age: -
Justification: The minimum time constraint for the origin of oxygenic cyanobacteria is usually based on the Great Oxidation Event (GOE), when the O_2 concentrations increased by several orders of magnitude in the Earth's atmosphere [101], which is considered as a result of oxygenic photosynthesis by oxygenic cyanobacteria [102,103]. However, some reported evidence for the rise of O_2 ~3000 Ma [104,105] based on geochemical and molecular records from the Pongola Supergroup, South Africa. This suggests that oxygenic photosynthesis thus oxygenic cyanobacteria might have appeared prior to GOE. Therefore, we used 3000 Ma as the minimum time constraint.
Alternatives: An alternative minimum age was set as 2320 Ma according to the estimated time of the GOE, which was used in the dating schemes *Wolfe 2019 (GOE)*, *Sánchez-Baracaldo 2014 (GOE),* and *Sánchez-Baracaldo 2017 (GOE)*. We alternatively set the time constraint, either 3000 Ma or 2320 Ma, on the crown group, rather than total group, of oxygenic cyanobacteria [dating schemes *Sánchez-Baracaldo 2017 (crown group)* and *Sánchez-Baracaldo 2017 (crown group+GOE)* in Supplementary Data 2], as many other studies did [91,92,100].

Node: Root (cyanobacteria-alphaproteobacteria split)
Minimum age: 3000 Ma
Maximum ages: from 3500 Ma to 4500 Ma with an interval of 100 Ma
Justification: We based the minimum age of the LCA of cyanobacteria and *Proteobacteria* on the minimum age of the total group of oxygenic cyanobacteria (3000 Ma) as described above. The maximum constraint is more difficult to determine because modern phylogenetics analysis showed that cyanobacteria likely have a very deep phylogenetic position whereas fossils of early life are often under controversy [106]. Therefore, we tried 11

different maximum ages by increasing the maximum age from 3500 Ma, when there were likely activities of early life [106–109], to 4500 Ma, which is approximately the age of Earth [110], with an interval of 100 Ma.
Alternatives: An alternative minimum age was set based on the GOE (2320 Ma), as described in the justification for the calibration on the oxygenic cyanobacteria total group. The alternative minimum age for the root was used in the dating schemes *Wolfe 2019 (GOE)*, *Sánchez-Baracaldo 2014 (GOE),* and *Sánchez-Baracaldo 2017 (GOE)*.

Supplementary Note 3: Additional discussion

S3.1. Limitations of the approach adopted in Shih *et al.*, 2017

Shih *et al.*, 2017 pioneered using the idea of endosymbiosis to date the evolution of cyanobacteria by analyzing 12 homologs shared by cyanobacteria, plastids, and mitochondria. However, since there are some cyanobacteria fossils well suited for molecular dating [92,111], it is unclear how much it improves the dating by integrating eukaryotic fossils. More importantly, mitochondria and plastids diverged long time ago, and both have undergone extensive gene reduction [17,112], which led to relatively few shared high-confidence orthologs. We found that five out of the 12 genes used by Shih *et al.*, 2017 were likely to contain large proportions of ambiguous sites in alignment (Supplementary Fig. 15), which could result in overestimates of the substitution rates of these genes, thus underestimates of divergence time. Possibly because of this issue, the origin times of the crown group of cyanobacteria estimated by Shih *et al.*, 2017 were at least 400 Ma younger than estimated by others [91,92,102]. Particularly, their estimated origin time of *Nostocale*s (~500 Ma) was much younger than the fossil records (before 1200 Ma [99,111]), although these fossils could be derived from stem group *Nostocales*.

S3.2. Limitations of calibrating the evolution of bacteria based on the divergence times of their modern hosts

Our time estimates of the divergences of *Alphaproteobacteria* were broadly consistent with the previous studies [29,113], but were considerably older than others [114–116]. The first two studies [29,113] calibrated the bacterial tree of life based on cyanobacteria fossils and/or other geologic records and biomarkers. In contrast, all of the latter three studies [114–116] based calibration bound constraints (in part) on a strict host-bacteria co-evolution. In other words, they assumed that pathogenic/symbiotic alphaproteobacterial lineages co-diverged with their modern hosts, and used the fossils of their modern hosts to calibrate the evolution of *Alphaproteobacteria*. However, none of them considered the possibility of host shifts in evolution, which, if indeed occurring, can lead to underestimates of the divergence times of bacteria.

Specifically, Chriki-Adeeb and Chriki, 2016 based their analysis on a single calibration. They assumed that *Rhizobium sullae* originated at the same time of their modern hosts, i.e., the Hedysaroid clade (Fabaceae: Hedysareae), 29.3 ± 3.0 Mya. However, host transitions within legumes are very frequent for rhizobia [117–120]. It is possible that the LCA of *R. sullae* established a symbiosis relationship with other legumes and had its hosts shifted later in evolution. This means that the symbiosis between *R. sullae* and Hedysaroid might have started after the divergence of *R. sullae*. If so, the use of the divergence time of the legume hosts (Hedysaroid) as the occurrence time of the LCA of *R. sullae* could lead to an underestimate of the origin time of *R. sullae* and of all analyzed alphaproteobacteria, as pointed out by Wang *et al.*, 2020. Consequently, the divergence time between *Alphaproteobacteria* and *Betaproteobacteria* estimated by Chriki-Adeeb and Chriki, 2016 was only 600 Ma.

Luo *et al.*, 2013 used three calibration points, two of which were based on fossil or geochemical evidence from cyanobacteria. As to the other one, it was based on the interaction between rhizobia and legumes, which was originally derived from an earlier

study [121]. Assuming that *Rhizobium* diverged from their non-rhizobia relatives after the appearance of legumes, Luo *et al.*, 2013 constrained the split of *Rhizobium* and its close relative (*Agrobacterium*) to be 120-100 Ma, the estimated origin time of legumes. This could be true if the LCA of *Rhizobium* indeed nodulated legumes. However, recent genomics studies showed that many *Rhizobium* strains, particularly the early-split lineages, do not adapt to a nodulating lifestyle, but are instead associated with diverse non-legume plants [27,122,123]. Hence, the origin time of legumes is plausibly more recent than the *Rhizobium-Agrobacterium* split. As a result, the assumption made in Luo *et al.*, 2013 (and originally in Ochman and Wilson, 1987) likely led to underestimates of the divergence times in the phylogeny.

Weinert *et al.*, 2009 dated the origin time of *Rickettsiales* to be 525-425 Ma. This study was based on the substitution rate of 16S rRNA. The substitution rate estimate was originally derived from an earlier study [124], where the authors assumed a co-divergence between *Buchnera* and its host (viz. aphids), and assigned the origin time of aphids as that of *Buchenera*. Similar to the study Chriki-Adeeb and Chriki, 2016, Weinert *et al.*, 2009 did not take into consideration the possibility of host shifts in evolution. Further, the substitution rate of the gene in *Buchnera*, which belongs to the *Gammaproteobacteria*, could be very different from that in *Rickettsiales*. Moreover, Weinert *et al.*, 2009 assumed a strict clock across the entire phylogeny. In other words, the authors assumed that non-*Rickettsiales* lineages, most of which are not parasitic bacteria, evolved at the same rate as *Rickettsiales*. Thus, this likely resulted in an overestimate of the substitution rate of non-*Rickettsiales* lineages, hence an underestimate of the divergence time between *Rickettsiales* and other alphaproteobacterial lineages.

S3.3. Implications for the evolution of *Rickettsiales*

Our understanding of *Rickettsiales* had long been limited to medically relevant members from the genera *Anaplasma*, *Ehrlichia*, *Neorickettsia* and *Rickettsia* [125], and to *Wolbachia*, which infect 20-75% of insect species and almost all filarial nematodes (reviewed in Bordenstein, 2003; Werren *et al.*, 2008). Thus, the predominant view, proposed by Weinert *et al.*, 2009, is that the LCA of *Rickettsiales* already had the ability to infect arthropods and shifted to other eukaryotes later ("animal first"). More recently, accumulating evidence indicates a much broader range of hosts of *Rickettsiales*, which expanded to annelids, sponges, cnidarians [128,129], green algae [130,131], and diverse protists particularly amoebae and ciliates [132–134]. An intriguing alternative hypothesis is therefore a "protist first" view where early *Rickettsiales* were associated with protists and switched to animals during evolution [135].

As detailed in Supplementary Note 3.1, the dated ages (525-425 Ma) of the crown group of *Rickettsiales* by Weinert *et al.*, 2009 , which have been widely cited by others [136–142], are likely to be largely underestimated due to methodological biases. Our revisited evolutionary timeline indicates that the LCA of *Rickettsiales* originated 1500 Mya, much earlier than the origin of animals but coincided with that of (unicellular) eukaryotes. Our results reject the view of the concurrence of the LCA of *Rickettsiales* and animals, and provide strong support to the "protist first" hypothesis. The reason is straightforward: if the parasites arose before the origin of their modern hosts, their interaction must be established later in parasites'

evolution. The dating result is reinforced by ancestral host reconstruction, which inferred a protist-associated LCA of *Rickettsiales* with a posterior probability of 96.5% and a substantially higher host transition rate from animals to protists compared to that from protists to animals (Fig. 2D-2E). Clearly, this points to independent host shifts from unicellular eukaryotes to animals, which, according to Fig. 2D, occurred once in the LCA of *Anaplasmataceae* whose all extant members are animal parasites, four times within the *Rickettsiaceae* where most members are associated with animal species, and three times in *Ca.* Midichloriaceae which are mostly associated with protists. Considering the diversity of *Rickettsiales* members yet to be studied and because the 16S rRNA genes used in our ancestral host reconstruction were only from representatives of each OTU, such evolutionary shifts are plausibly more frequent than here estimated. Note that a recent study described a new *Rickettsiales* family *Deianiraea*, which were reported to be attached to the surface of the ciliate *Paramecium* but never invading the inside of the host cell [143]. This opens a possibility that the LCA of *Rickettsiales* adapted to an extracellular parasitic lifestyle or was a facultatively intracellular bacterium. Nevertheless, it is clear that interaction between protists and *Rickettsiales* has a prominent role in *Rickettsiales* evolution and diversification.

Rickettsiales inhabiting hematophagous arthropods are transmitted to humans by either the bite of infected mites and ticks or the feces of infected lice and fleas that are inhaled or rubbed into the skin [144], which, however, cannot be the case for those inhabiting protists. It is hypothesized that protist-harboring *Rickettsiales* could be transmitted to animals sharing the same habitat via the feeding behaviour of the animal host, which presumably happened in aquatic environments where a variety of protists share the same habitats with invertebrates with grazing- or filter-feeding strategies [135,145]. This view is further supported by the recent finding of the transfer of a *Rickettsiales* bacterium (*Ca.* Trichorickettsia mobilis) to a metazoan (the planarian *Dugesia japonica*) from an infected ciliate *Paramecium* in the same aquatic environment [146]. It would be important to further investigate the diversity of *Rickettsiales* hosted by protists and how emerging pathogens might arise from there. In addition, comparative genomics studies between phylogenetically closely related *Rickettsiales* with different hosts may help identify virulence genes and provide clues to targets for vaccine development.

S3.4. Selection of the clock model

It is important to note that the selection of the clock model, among others, had a substantial impact on the posterior ages for both the mito- and nuclear-encoded datasets. The estimated divergence times of the *Rickettsiales* and mitochondrial lineages were consistent between using the AR and IR model implemented in MCMCTree. The IR model inferred a long branch leading to the divergence of non-*Rickettsiales* alphaproteobacteria (Supplementary Fig. 4F). Consequently, the estimated posterior ages of all non-*Rickettsiales* lineages were considerably smaller if inferred with the IR model.

The analysis with mcmc3r, however, showed that the AR model outperformed the IR model, as indicated by their higher marginal likelihoods (Supplementary Fig. 12A). Thus, the AR model likely better explains the rate variation across lineages in our data, and was therefore used as the preferred model. This idea is further supported by the following lines of evidence. First, molecular clock dating infers not only divergence times but also the

substitution rate of each lineage [147]. It is well known that *Rickettsiales* and mitochondrial lineages display higher evolutionary rates than other alphaproteobacteria [12,16,17,148]. The rate differences were well estimated by the AR model but not the IR model, since under the IR model the substitution rate differences between *Rickettsiales* and non-*Rickettsiales* alphaproteobacteria, and between mitochondria and non-*Rickettsiales* alphaproteobacteria were very small (Supplementary Fig. 13B, 13C). Second, the result obtained using PhyloBayes was more consistent with that obtained with the AR model than the IR model using MCMCTree (Supplementary Fig. 3). Further research is necessary to better appreciate the merits of each model in modelling the evolutionary processes on different time scales and of different species [21,149].

Supplementary Figure 1. Phylogenomic reconstruction of *Alphaproteobacteria* and mitochondria, and presentation of alternative topologies which differ in the phylogenetic positions of *Rickettsiales*, *Holosporales*, *Pelagibacterales*, and mitochondria. (A, B) The trees were generated using IQ-Tree under the C30 profile mixture model with PMSF approximation based on the dayhoff4 (A) and SR4 (B) recoding scheme, respectively. Circles in black and grey on the nodes denote nodes with the IQ-Tree ultrafast bootstrap values ≥ 95% and ≥ 85%, respectively. The scale bars denote 0.05 substitutions per amino acid site. The phylogeny shown in panel A was used in the main analysis for dating (with more eukaryotes added to the mitochondria subtree [see Supplementary Figure 2A and Supplementary Note 1.1]). (C) The 12 different topologies of the *Alphaproteobacteria* phylogeny used in dating. *Topo1* represents the one shown in panel A.

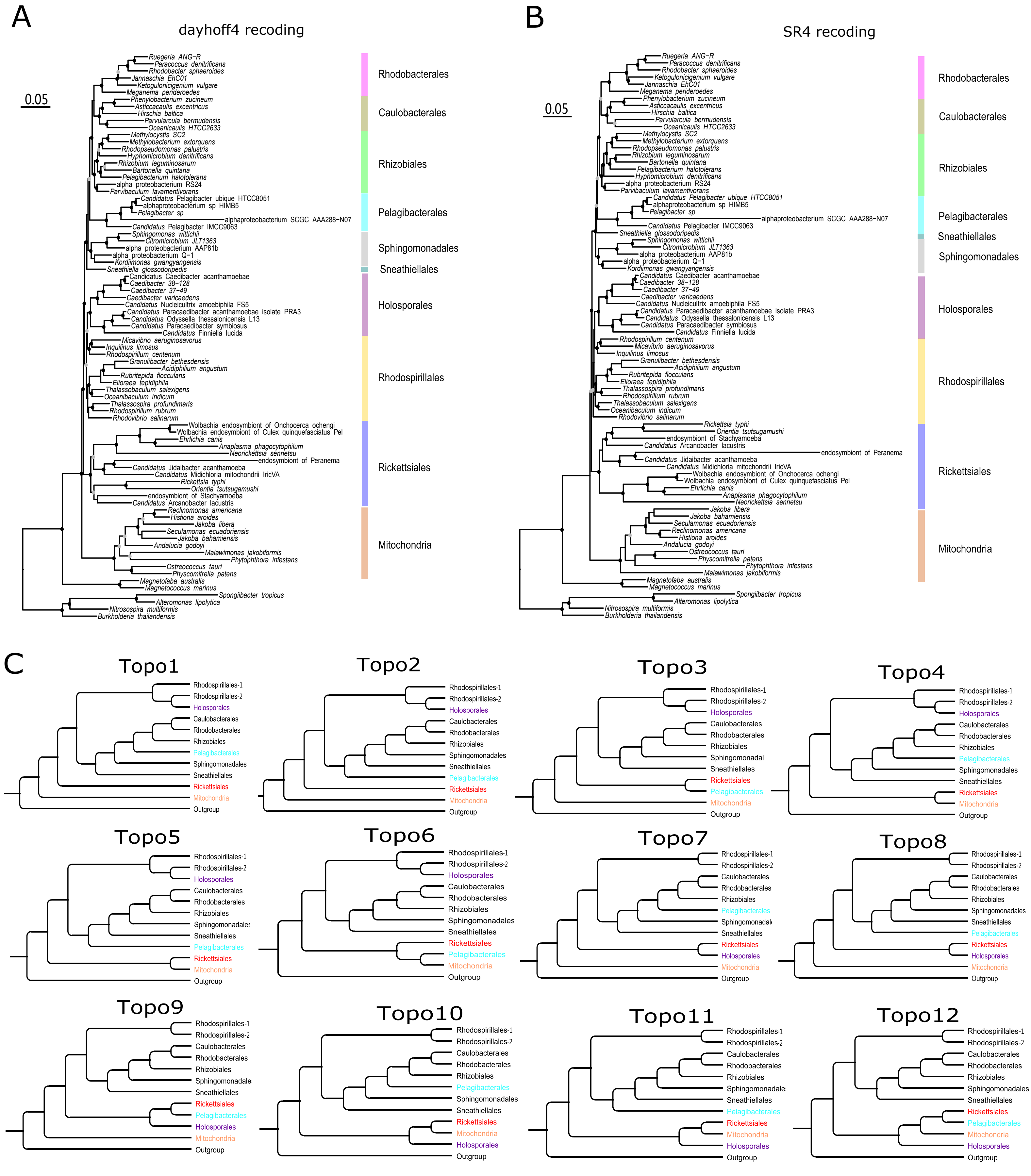

Supplementary Figure 2. Phylogenetic positions and calibration densities of calibration points for the mitochondria-based (A) and cyanobacteria-based (B) strategy. (A) Calibration information of all of the ten nodes were used in the dating analysis of the nuclear-encoded dataset. Only Nodes 1-4 were used for the mito-encoded dataset. The calibrations are detailed in Supplementary Note 2.1. Tips labelled with dark green and purple circles indicate those used in both datasets and only the nuclear-encoded dataset, respectively. Tips with a light green circle denote those used only in the mito-encoded dataset due to the unavailability of their nuclear genome sequences. The topology is based on general understanding of the eukaryotic phylogeny (see Supplementary Note 1.1). (B) All of the three calibration points are involved in the best-practiced dating scheme (but not necessarily in alternative dating schemes) for cyanobacteria-based dating. The tree topology is according to IQ-Tree phylogenomic reconstruction. All species present in the tree were used in the cyanobacteria-based dating. Shown on the right panel are the calibration densities for the calibration points (the user-specified probability densities at which the clade originated), according to the best-practiced dating scheme. In brief, a uniform distribution is applied to the calibration points where both minimum and maximum bounds are available. For nodes 1-3 in the cyanobacteria-based dating, because only a minimum bound is available, a Cauchy distribution is applied (see Supplementary Note 2.2).

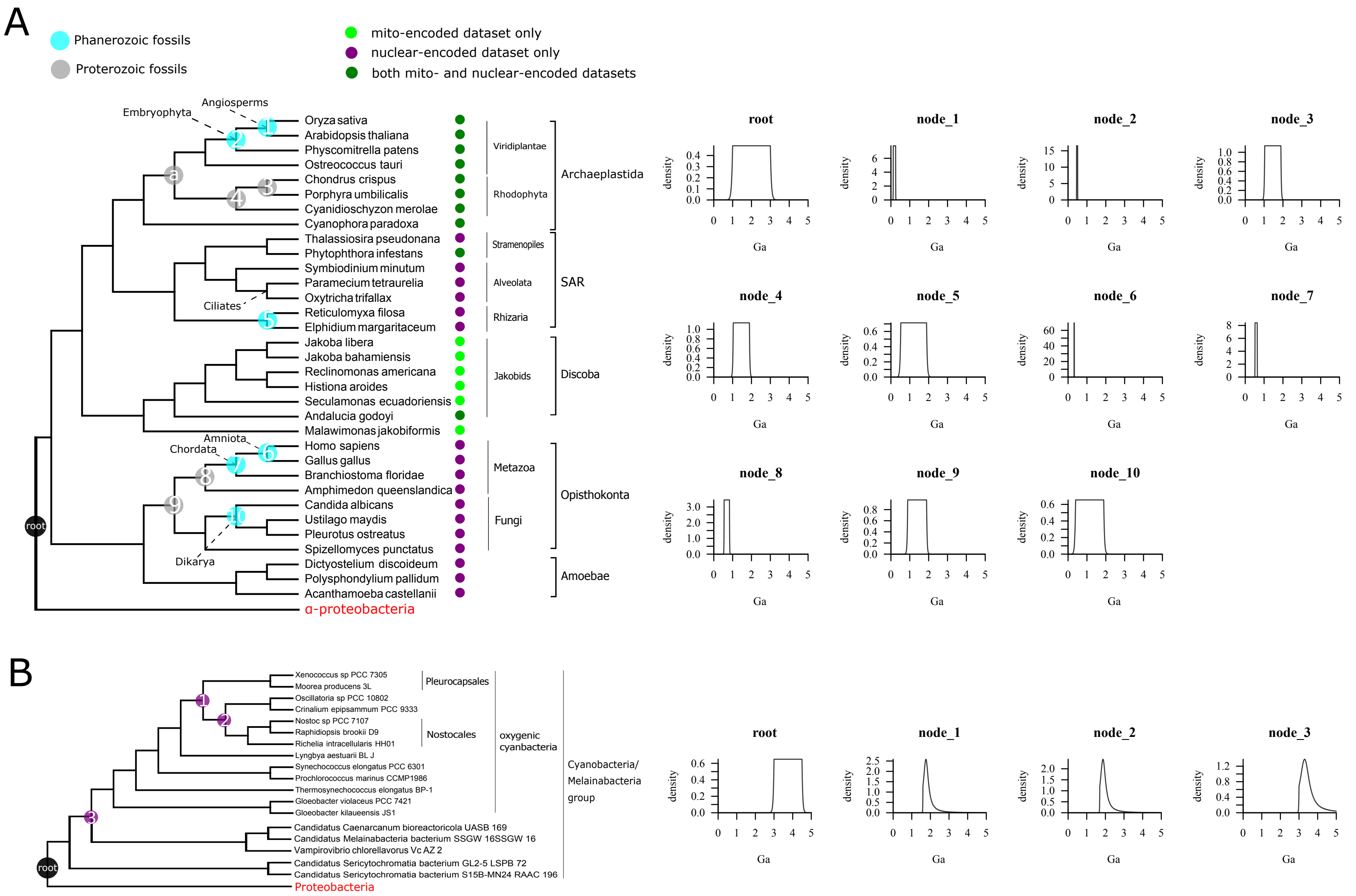

Supplementary Figure 3. Comparison of the chronograms obtained using PhyloBayes with amino acid sequences or MCMCTree with nucleotide sequences and the best-practiced dating scheme for mito- and nuclear-encoded datasets. Nodes are drawn at the posterior means. (A) PhyloBayes with amino acid sequences and the log-normal autocorrelated clock model ("-ln"). (B) PhyloBayes with amino acid sequences and the uncorrelated gamma multipliers ("-ugam"). (C) MCMCTree with coding sequences (only the first two positions of a codon). (D) MCMCTree with amino acid sequences based on the best-practiced scheme.

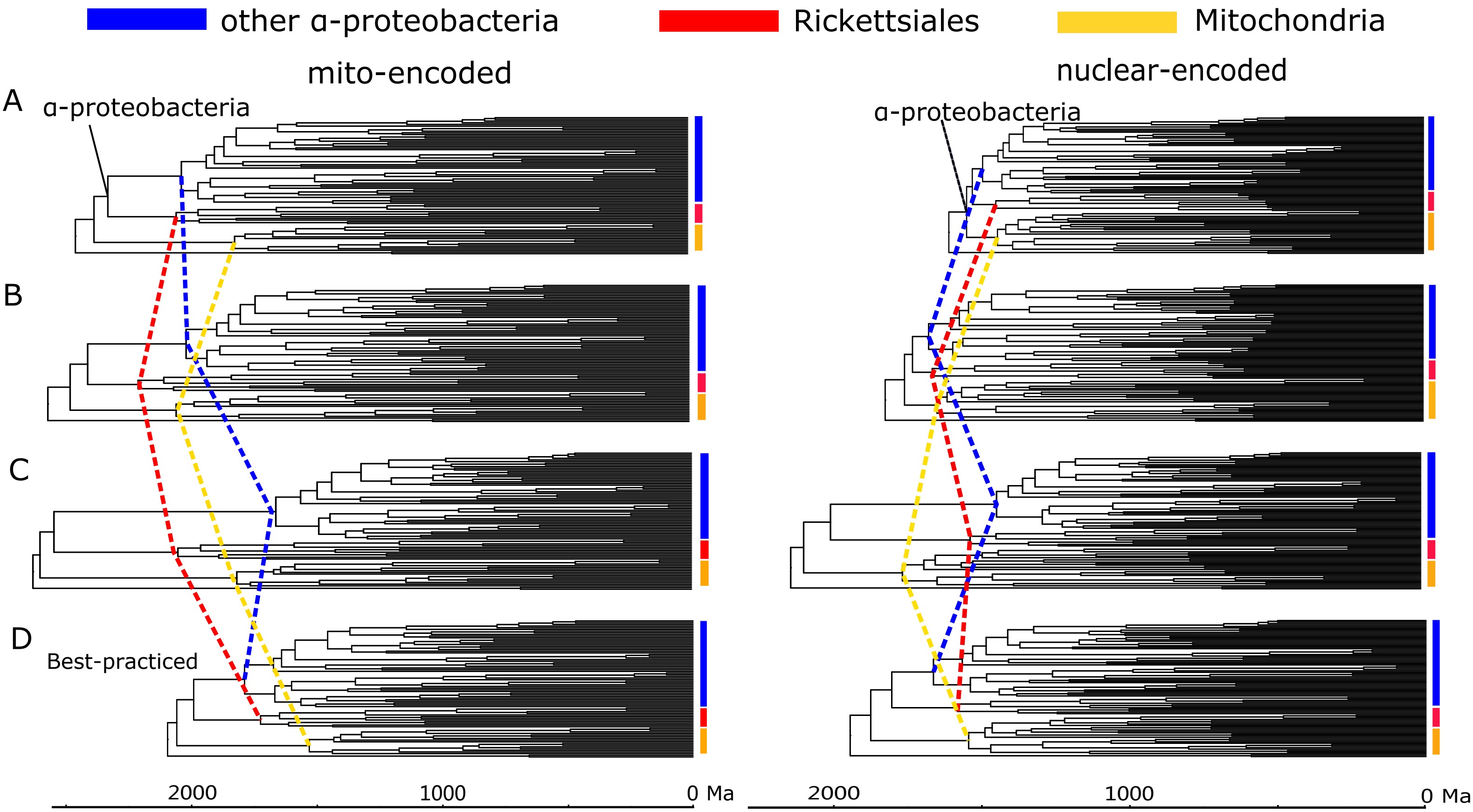

Supplementary Figure 4. Comparison of the chronograms obtained with different dating schemes using MCMCTree for mito- and nuclear-encoded datasets (detailed calibrations are given in Supplementary Data 2). *Phan*: only calibration points with Phanerozoic fossils considered. *Max-1*: maximum constraints for nodes with controversial maximum ages removed. *Max-2*: maximum constraints for nodes whose maxima are set as 1891 Ma based on the earliest eukaryotic fossils removed. *Single partition*: all sequences considered as a single partition. *STR*: strict rates clock model. *IR*: independent rates clock model.

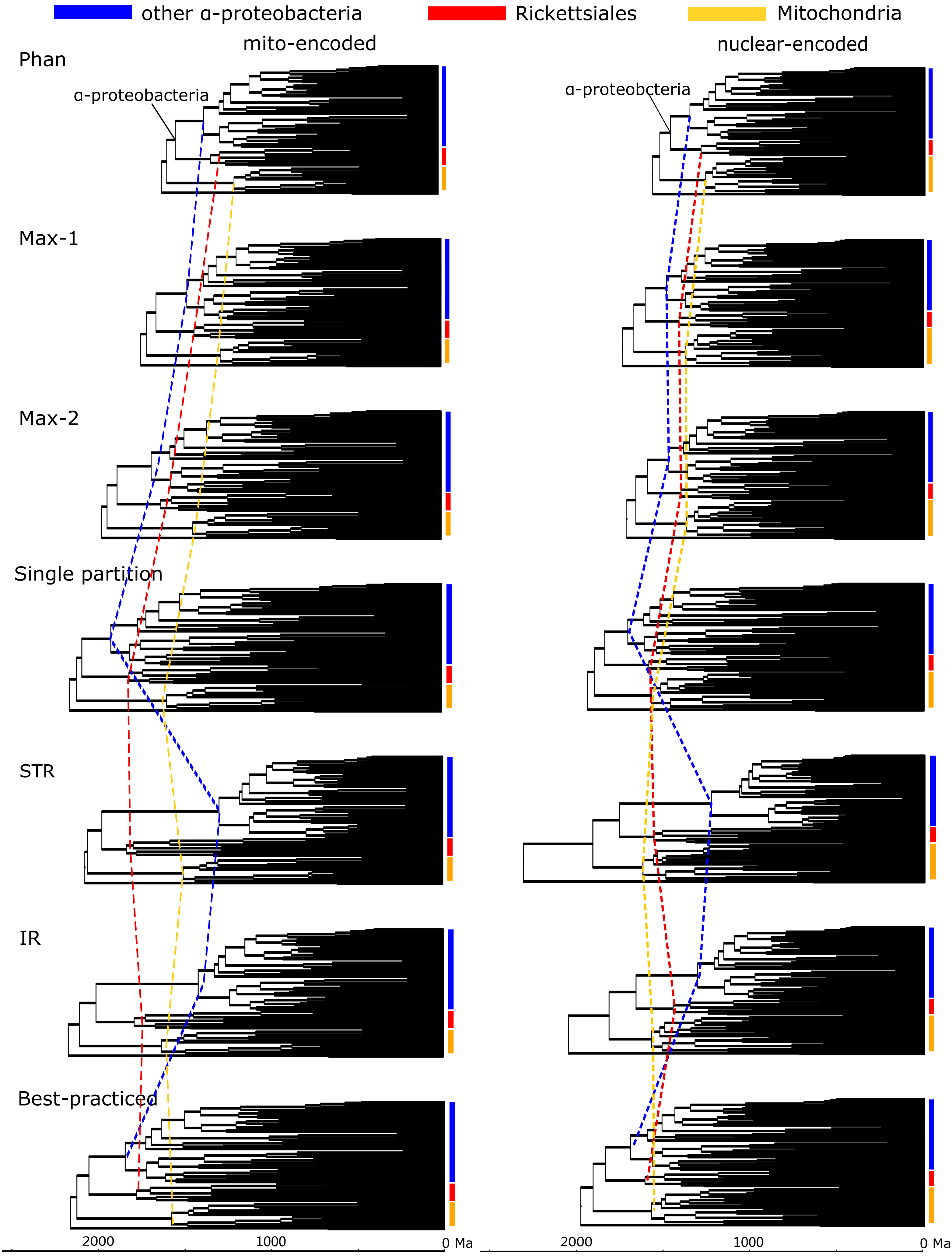

Supplementary Figure 5. The divergence times estimated using alternative schemes (y-axis) versus using the best-practiced scheme (x-axis) using MCMCTree for the mito- and nuclear-encoded datasets (see Supplementary Data 2 for details). *Root-1*: Root age calibrated as 3500-1000 Ma. *Root-2*: Root age calibrated as 4000-1000 Ma. *Root-3*: Root age calibrated as 4500-1000 Ma. *No max*: maximum constraints on all internal calibration points removed, leaving only maximum age of 3000 Ma on the root. *Sigma*: the prior of the standard deviation of log rate on branches was set to 1.0 to inform very large rate variation among branches.

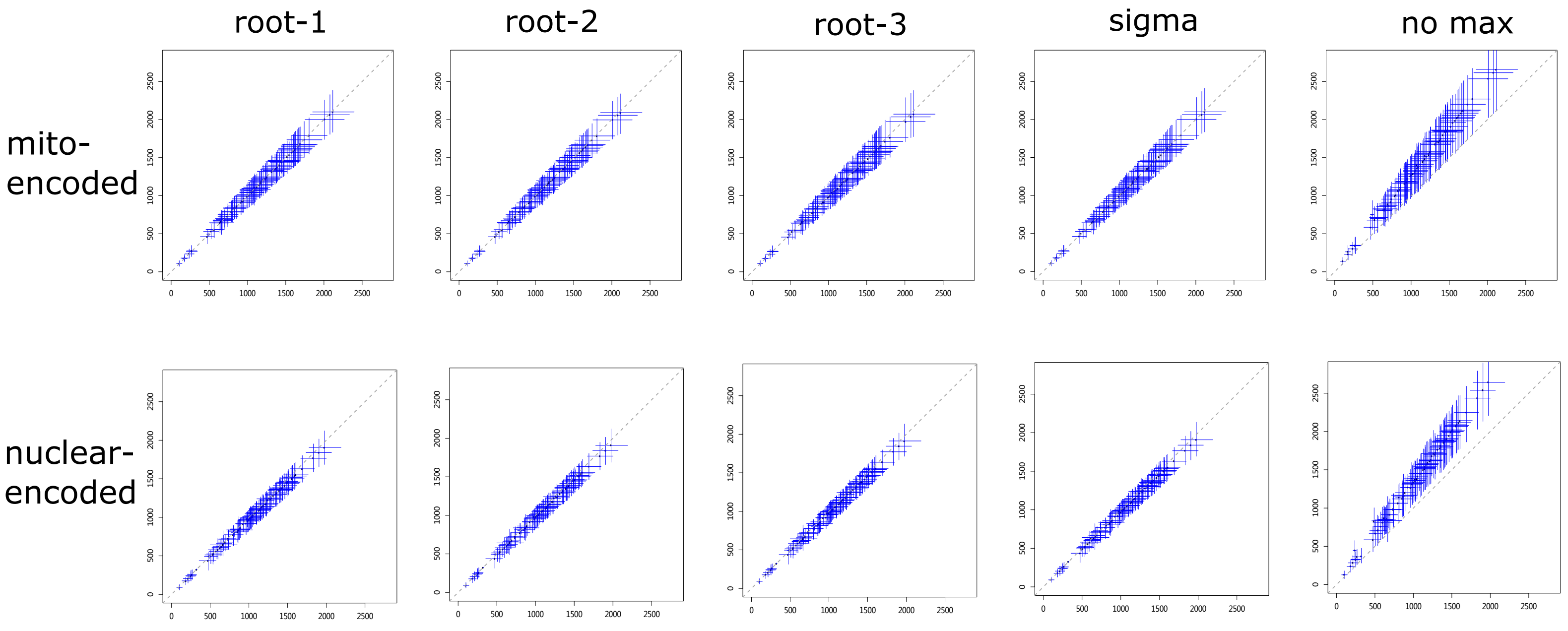

Supplementary Figure 6. The divergence times (A) and infinite-sites plots (B) estimated with different sequence partitioning strategies in MCMCTree analysis for the mito-encoded and nuclear-encoded datasets. (A) The divergence times estimated using different numbers of partitions (y-axis) versus using the best-practiced scheme (fully partitioned) with MCMCTree. ModelFinder partitions indicate partitioning based on ModelFinder. Details of the partitioning are given in Supplementary Data 2 and the online repository Figshare (see Data availability). (B) Infinite-sites plots obtained by using different numbers of partitions. The uncertainty in the divergence time, measured as the 95% HPD width, is plotted against the posterior mean of times for each node. A lower value of the slope indicates less changes in the 95% HPD width, hence higher precision in dating. The infinite-sites plots for the best-practiced dating scheme are displayed in Fig. 1.

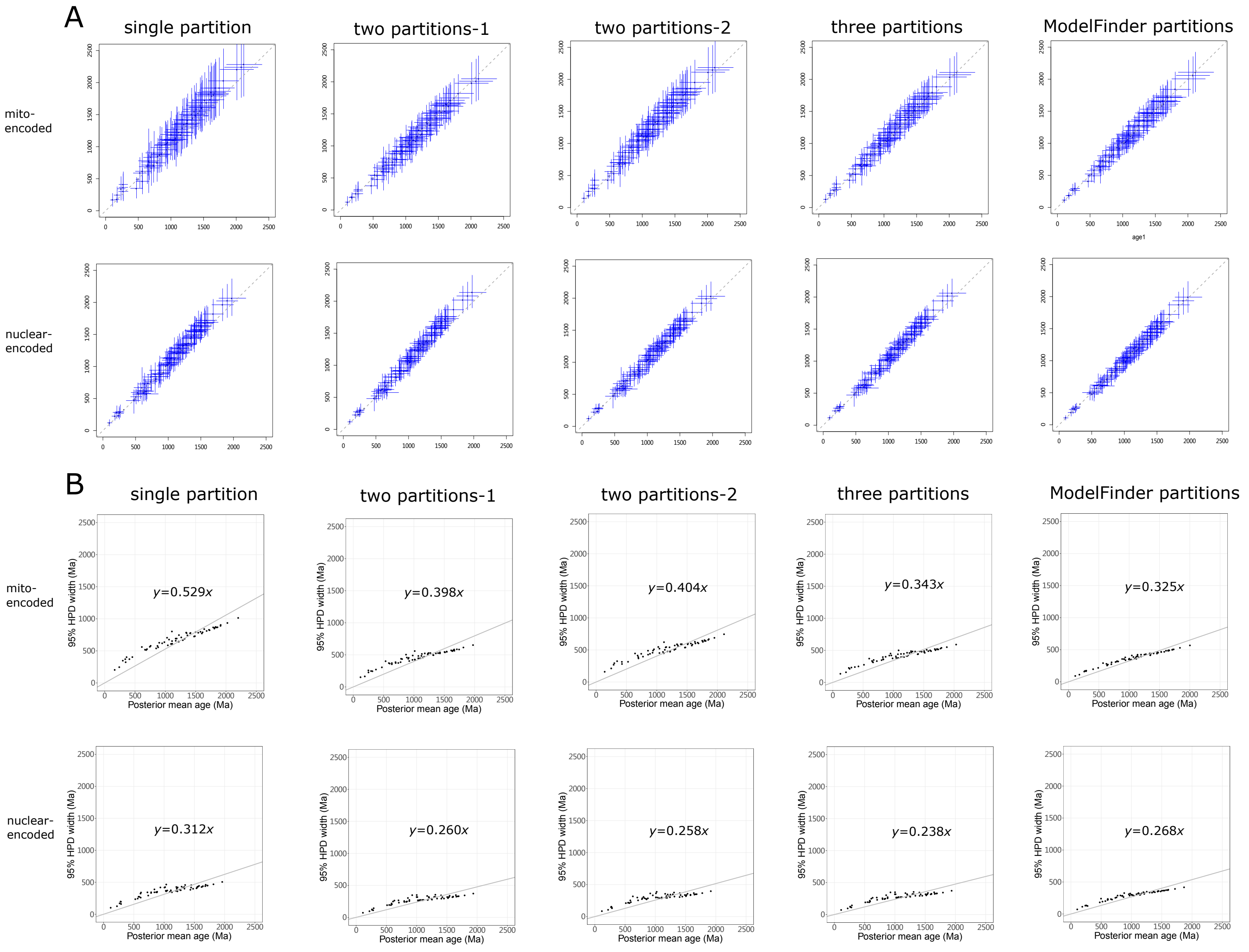

Supplementary Figure 7. The composite chronograms combining uncertainties associated with Bayesian relaxed molecular clock analysis including calibrations, models, and partitions for the mitochondria-based strategy. The node ages and the 95% HPD intervals were derived from the composite of the posterior estimates across six competing dating schemes (the best-practiced scheme together with the five alternatives shown in Fig. 2A, namely *Phan*, *Max-1*, *Root-1*, *Single partition*, and *IR*). Node bars indicate 95% HPD intervals of posterior ages. (A) The mito-encoded dataset. (B) The nuclear-encoded dataset.

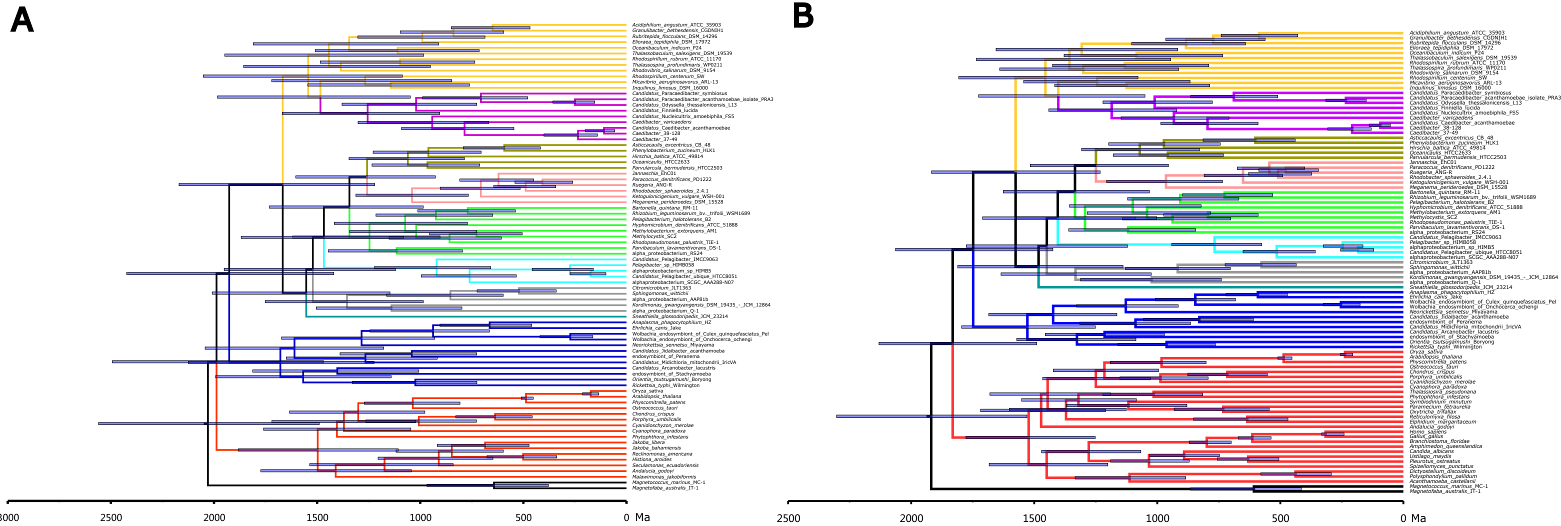

Supplementary Figure 8. MCMCTree analysis with an expanded genome set. (A) Chronograms obtained using the expanded genome set. Sixteen MAGs from *Alphaproteobacteria* reported by Martijn et al., 2018 (tips highlighted in red) are used in addition to the genomes used in the main analysis. (B) Comparison of the divergence times (95% HPD interval) of major clades estimated by using the original genome set and the expanded genome set. The center corresponds the posterior mean age.

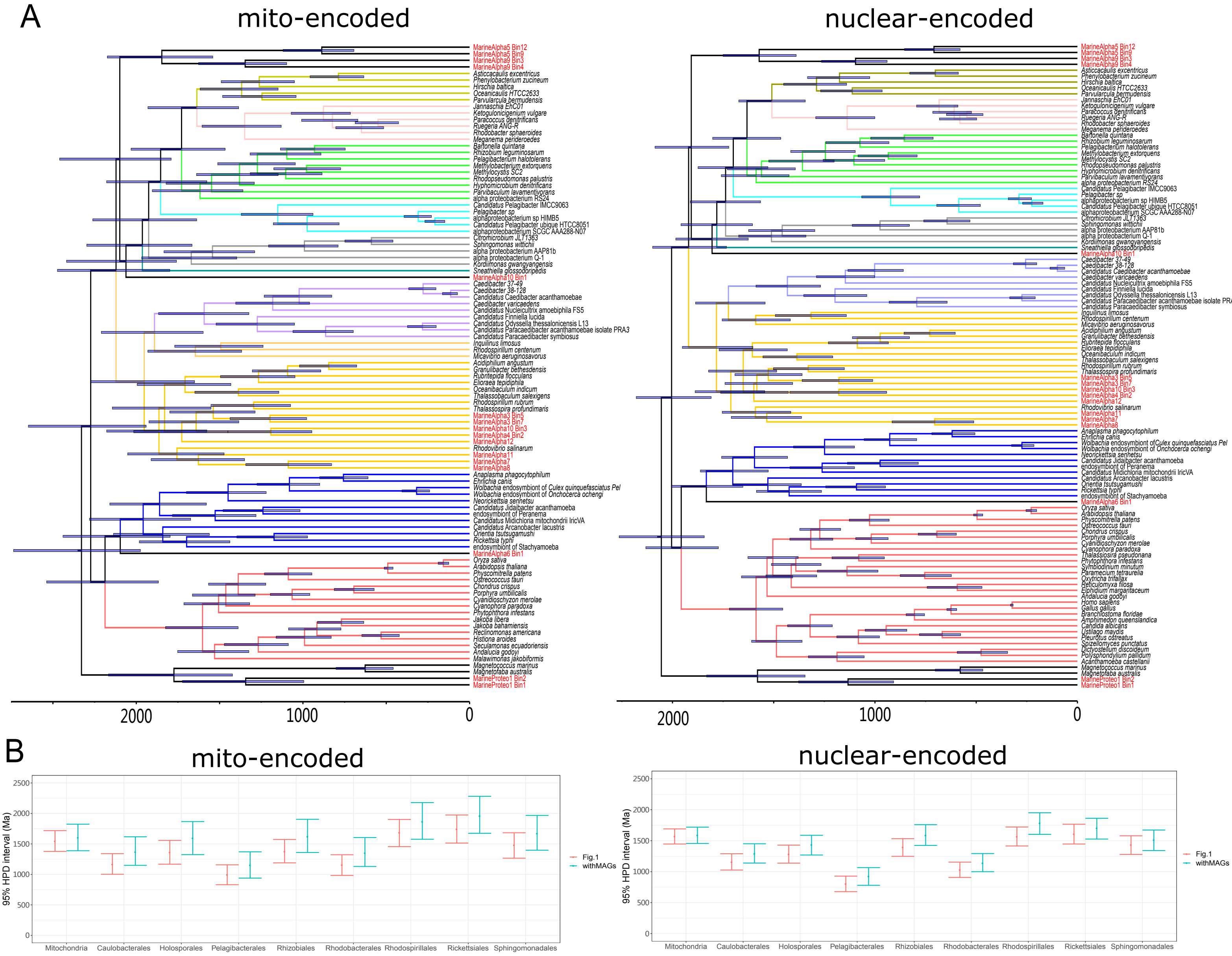

Supplementary Figure 9. Time estimates by co-estimating both tree topology and divergence times with BEAST. (A) Chronograms obtained using BEAST. (B) Comparison of the divergence times (95% HPD interval) of major clades estimated by using MCMCTree and BEAST respectively. The center corresponds the posterior mean age.

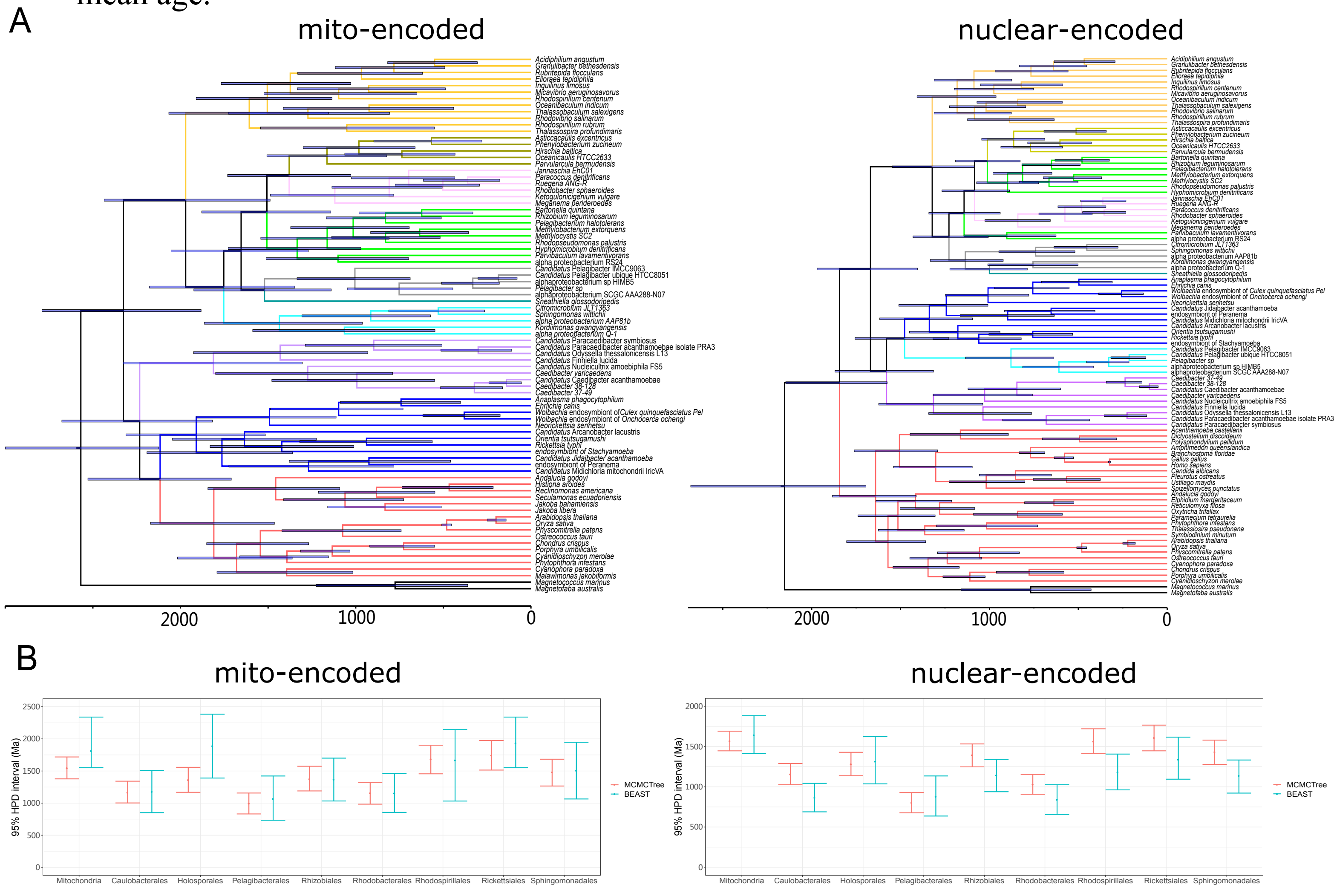

Supplementary Figure 10. Comparison of the chronograms obtained with different topologies of the mitochondria subtree (shown in the left panel) using MCMCTree for the nuclear-encoded dataset.

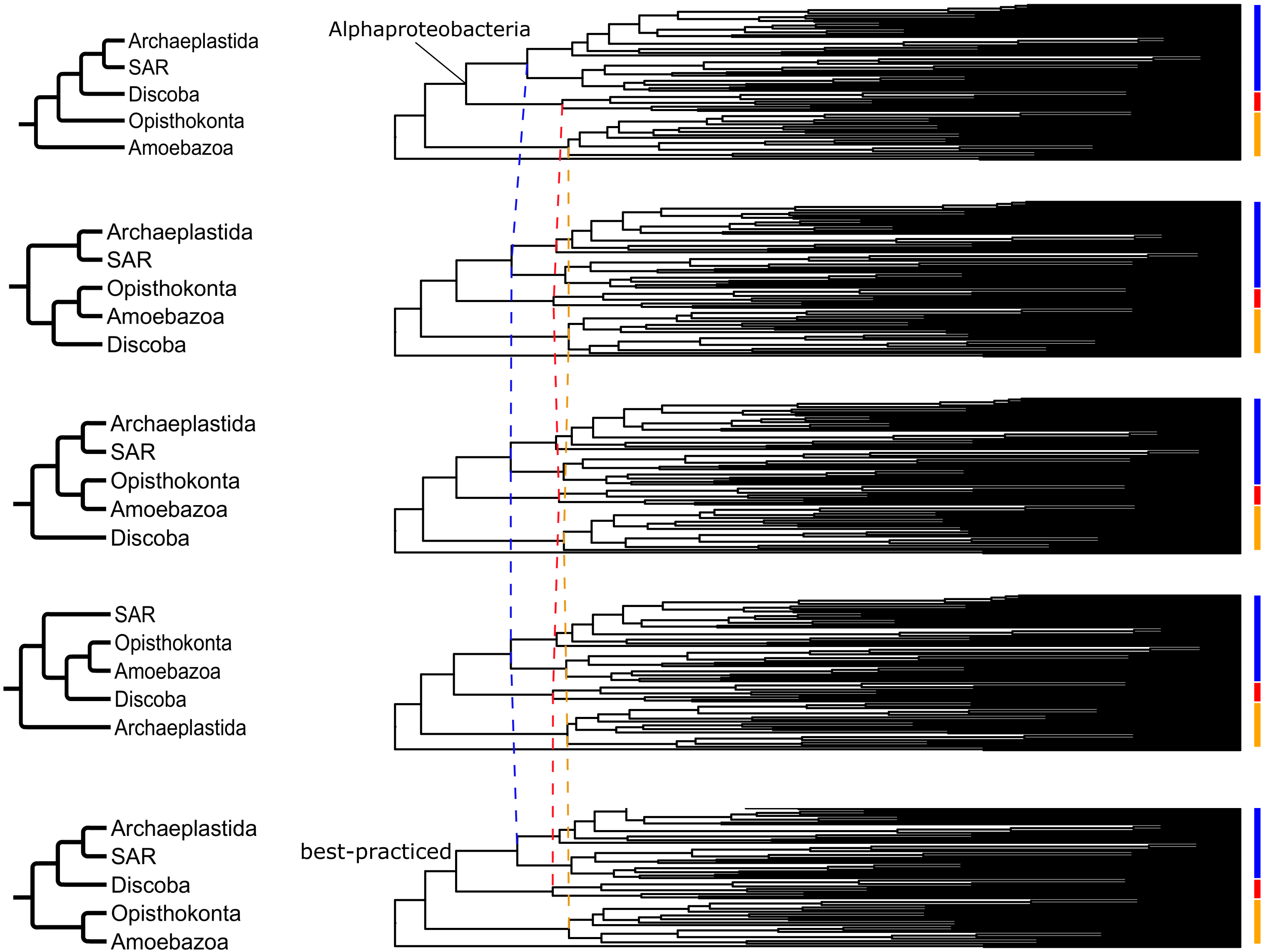

Supplementary Figure 11. Posterior ages of major clades estimated based on genes of different rate categories. Genes are classified into different categories based on their relative difference in the average substitution rate estimated by MCMCTree between mitochondria and non-*Rickettsiales Alphaproteobacteria*. The relative difference is calculated as $\frac{|rate[mito] - rate[\alpha]|}{\max(rate[mito],\ rate[\alpha])}$ [150]. Genes in each rate category are those with a relative difference in rate smaller than the cutoff (from 0.2 to 0.7). Genes with a smaller value of the rate relative difference may show more similar evolutionary rates between mitochondria and *Alphaproteobacteria*. The numbers in the parentheses mean the number of genes included each category. The y-axis indicates the posterior mean age and corresponding 95% HPD interval.

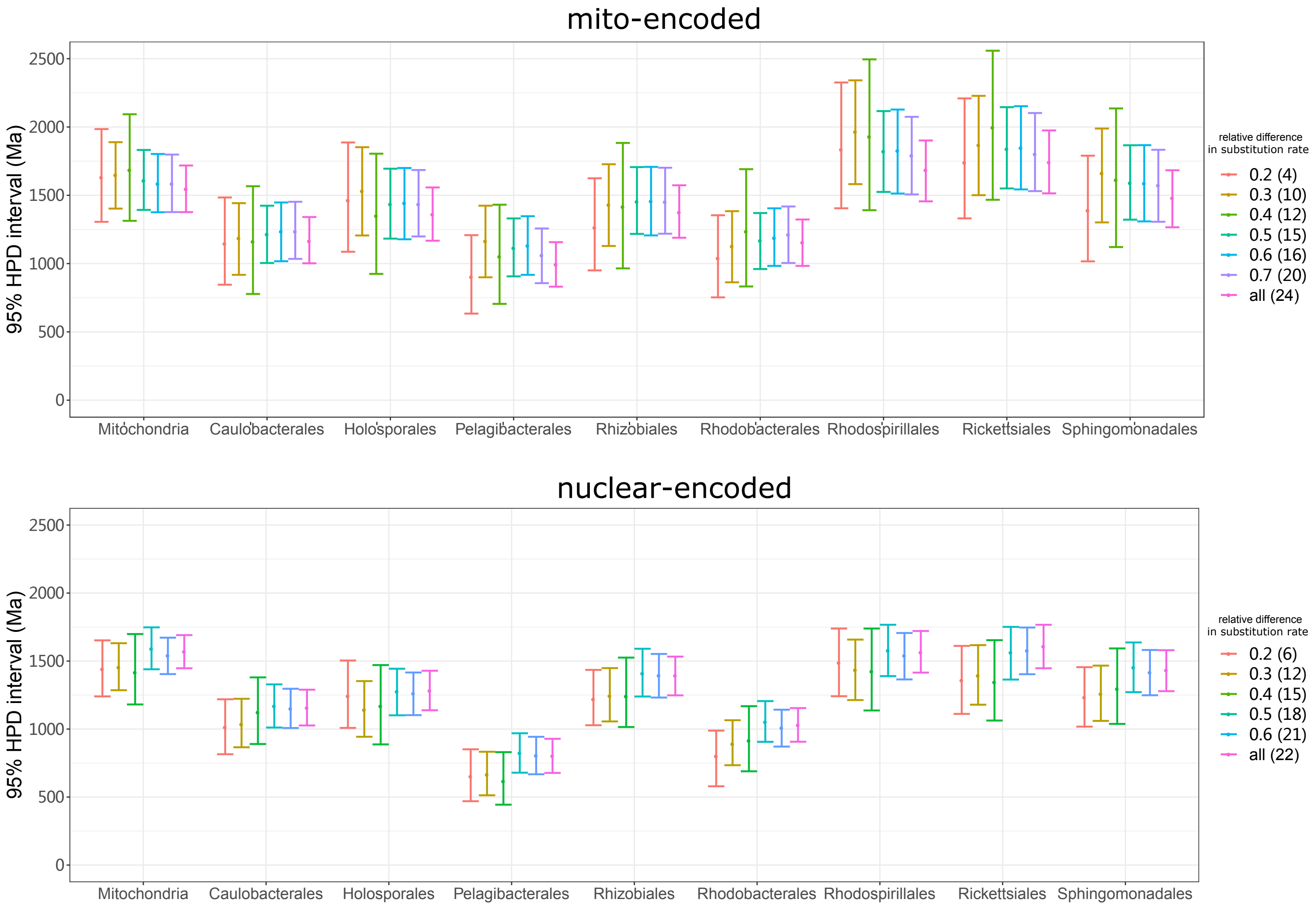

Supplementary Figure 12. Comparison of the posterior mean ages for alphaproteobacterial lineages estimated using the mitochondria- and cyanobacteria-based strategies. (A) Plots showing the posterior mean ages of major clades estimated under root calibrations from 3500 to 4500 Ma (with an interval of 200 Ma) for the cyanobacteria-based strategy based on eight different dating schemes (Supplementary Data 2). *Sánchez-Baracaldo 2017-1* is also used in Fig. 2C. (B) Posterior mean ages estimated with the cyanobacteria-based (y-axis) and mitochondria-based methods (x-axis). The cyanobacteria-based method is based on the dating scheme *Sánchez-Baracaldo 2017-1* (Supplementary Data 2) with different root maximum constraints from 3500 Ma to 4500 Ma. The mitochondria-based method is based on the best-practiced scheme of the mito-encoded dataset (Supplementary Data 2).

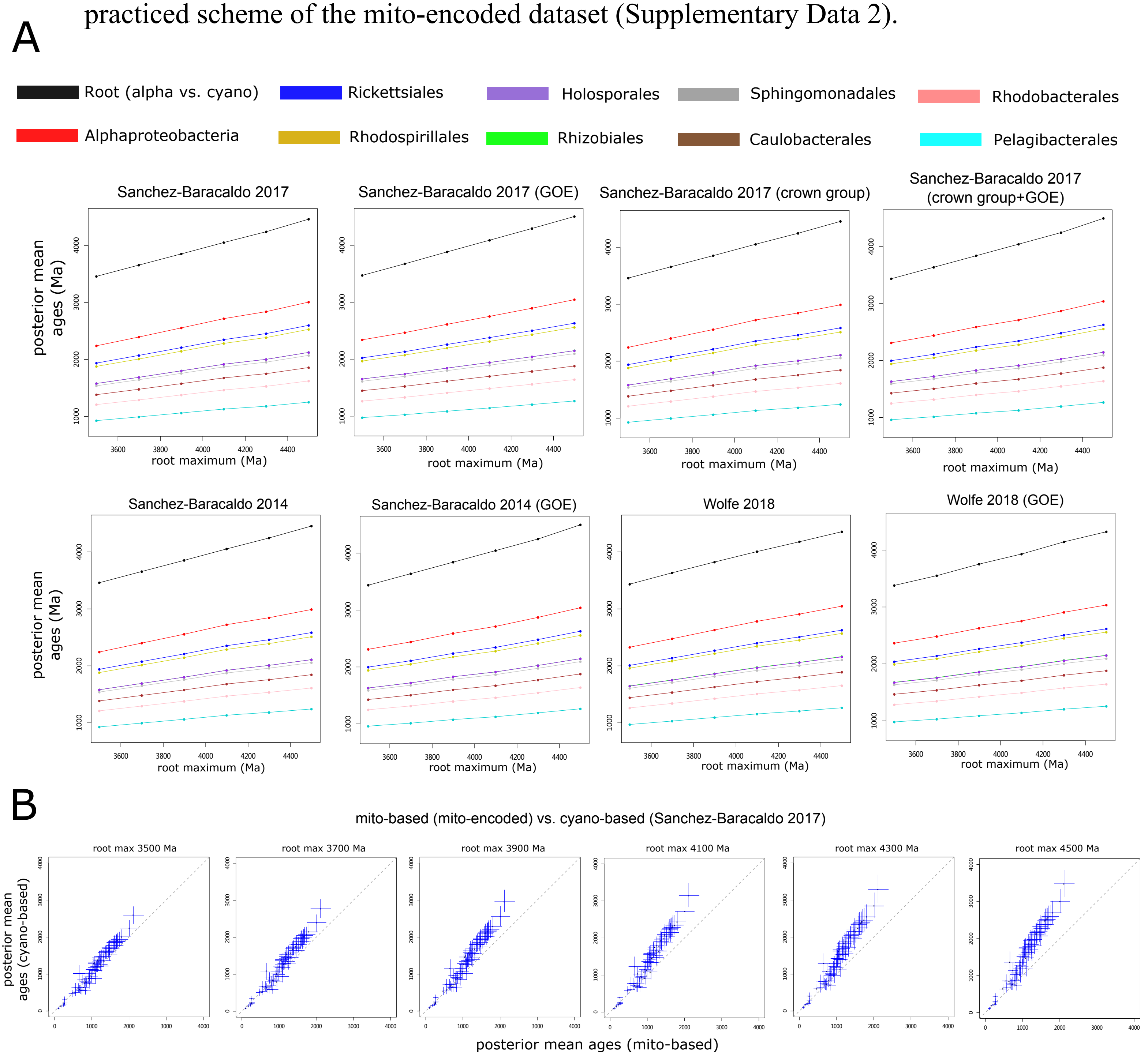

Supplementary Figure 13. Selection of the best-fit clock model in MCMCTree analysis. (A) Results of mcmc3r analysis. The number of genes (g) and species (s) for each dataset are indicated. The tested clock models are the autocorrelated rates clock (AR), independent rates clock (IR), and the strict clock (STR). *Pr* indicates the posterior probabilities of different clock models, and those higher than 0.75 correspond to substantial evidence according to the evidence categories for the Bayes Factor given by Jeffreys, 1961. The model with the highest posterior probability (and above 0.75) is shown in bold type. The data for mcmc3r analysis are available at the online open access repository FigShare (see Data and code availability). (B, C) Boxplots of the average substitution rates (amino acid changes per site per 100 million years) of branches belonging to the mitochondria, *Rickettsiales* and other alphaproteobacterial clades using the mito-encoded (B) and nuclear-encoded (C) dataset. The bottom and top of the box correspond to the first quartile (Q1) and third quartile (Q3) respectively. The middle bar indicates the median. Whiskers represent the 1.5 interquartile range (IQR). The substitution rates were estimated by MCMCTree under the AR and IR model, respectively. The *P*-values were calculated using a two-sided paired t-test (*n*=10 mito-encoded dataset, *n*=22 nuclear-encoded dataset).

A

	Mito-encoded			Nuclear-encoded		
Data	Model	Log ML	Pr	Model	Log ML	Pr
20s, 3g	**AR**	-29563.91	0.861	AR	-24155.84	0.072
	IR	-29565.73	0.139	**IR**	-24153.29	0.928
	STR	-29697.46	0	STR	-24284.6	0
20s, 5g	**AR**	-53950.53	0.781	**AR**	-37694.31	0.974
	IR	-53951.8	0.219	IR	-37697.95	0.026
	STR	-54225.71	0	STR	-37886.26	0
20s, 10g	**AR**	-91982.55	0.999	AR	-97673.1	0.468
	IR	-91989.26	0.001	IR	-97672.97	0.532
	STR	-92322.99	0	STR	-98195.11	0
40s, 3g	**AR**	-35186.76	1	**AR**	-32660.08	0.879
	IR	-35196.25	0	IR	-32662.06	0.121
	STR	-35412.79	0	STR	-32792.46	0
40s, 5g	**AR**	-81426.1	0.988	**AR**	-65955.1	1
	IR	-81430.48	0.012	IR	-65966.05	0
	STR	-81923.47	0	STR	-66271.04	0
40s, 10g	AR	-156821.1	0	AR	Not converged	
	**IR**	-156805.5	1	IR	-126709.4	N/A
	STR	-157618.8	0	STR	-127333.6	

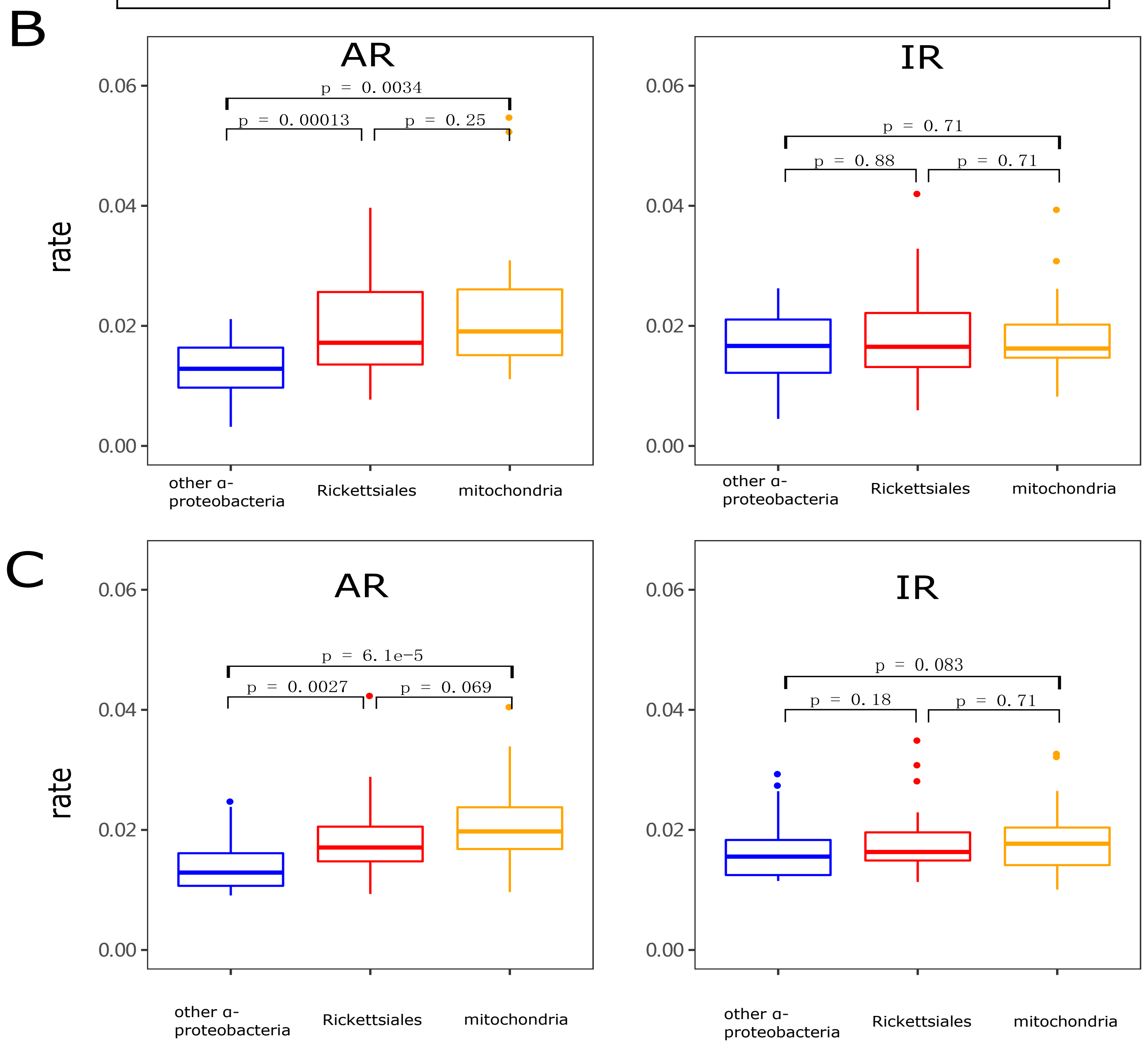

Supplementary Figure 14. Reconstruction of ancestral hosts of *Rickettsiales* and transition rates between animal- and protist-associated lifestyles with randomly selected representatives from each OTU (defined by 98.7% identity of 16S rRNA gene). (A) Inferred ancestral hosts of *Rickettsiales*. The pie charts on the nodes show the estimated probabilities of the hosts, and the branch colors indicate the hosts with the higher probability at the corresponding nodes. (B) The transition rates from animal-associated to protist-associated (q_{AP}) and from protist-associated to animal-associated (q_{PA}) estimated by the MCMC method in BayesTraits multistate. The log-transformed Bayes factor (logBF) used to compare between different models is shown, where values above 10 are considered very strong evidence for support [48]. The sources and credits of the cartoon graphs are provided in the online open access repository FigShare (see Data and code availability).

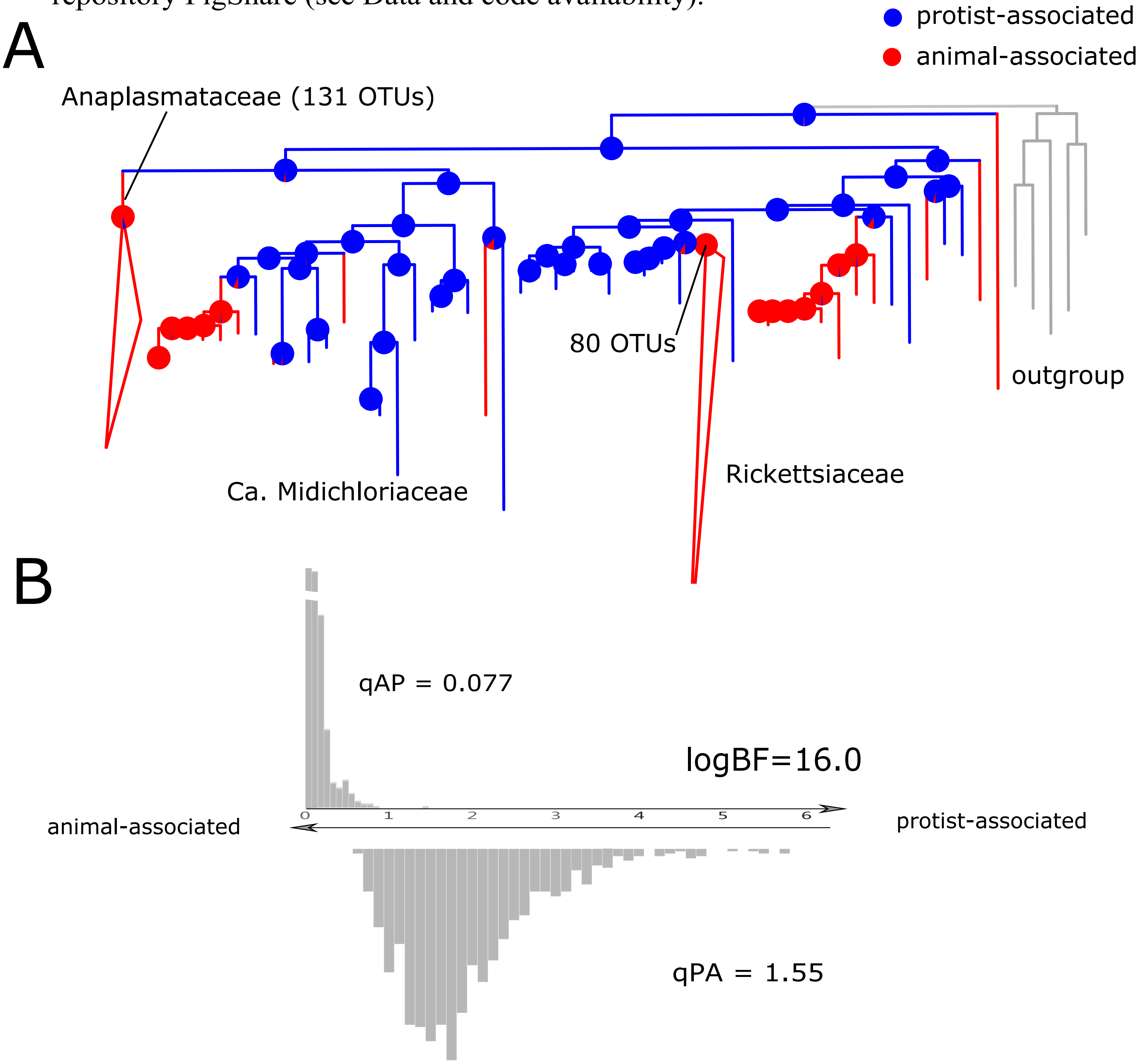

Supplementary Figure 15. Visualization of the multiple sequence alignments of the five protein-coding genes that likely contain large proportion of ambiguous sites across bacteria, plastids and mitochondria analyzed in Shih *et al.*, 2017. The alignments were generated using MAFFT v7.222 with default settings. Amino acids are shaded according to the color scheme based on the *BLOCUM62* substitution matrix. (A) *AtpE*. (B) *AtpH*. (C) *AtpF*. (D) *Rpl2*. (E) *Rps3*.

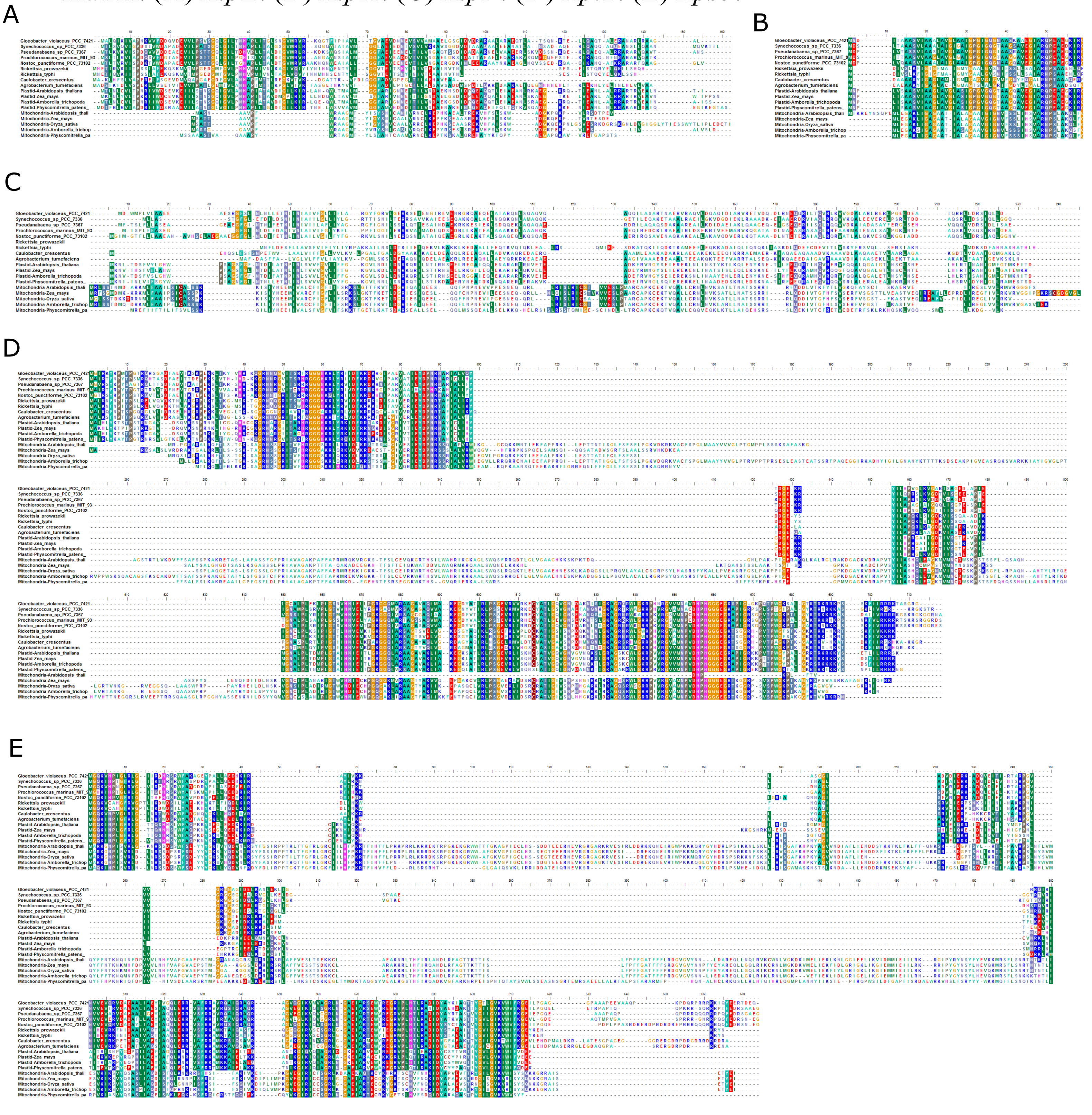

Supplementary Figure 16. Visualization of the multiple sequence alignments of the six protein-coding genes that do not contain large proportion of ambiguous sites across bacteria, plastids and mitochondria analyzed in Shih *et al.*, 2017. The alignments were generated using MAFFT v7.222 with default settings. Amino acids are shaded according to the color scheme based on the *BLOCUM62* substitution matrix. (A) *AtpA*. (B) *AtpB*. (C) *AtpI*. (D) *EF-Tu*. (E) *Rpl16*. (F) *Rps12*.

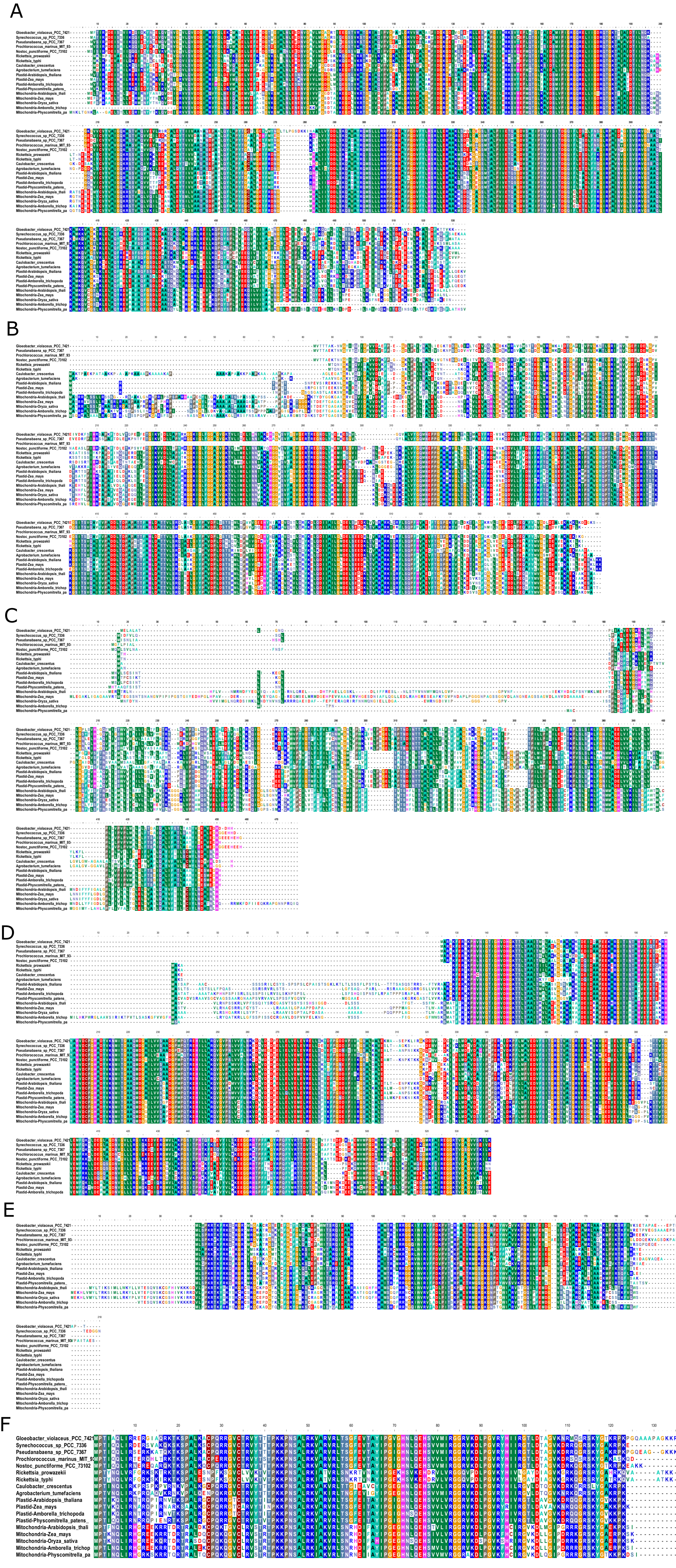

References

1. Shih, P. M., Hemp, J., Ward, L. M., Matzke, N. J. & Fischer, W. W. Crown group Oxyphotobacteria postdate the rise of oxygen. *Geobiology* **15**, 19–29 (2017).
2. Howe, K. L. *et al.* Ensembl Genomes 2020-enabling non-vertebrate genomic research. *Nucleic Acids Res.* **48**, D689–D695 (2020).
3. Bateman, A. UniProt: A worldwide hub of protein knowledge. *Nucleic Acids Res.* **47**, D506–D515 (2019).
4. Basu, S. *et al.* DictyBase 2013: Integrating multiple Dictyostelid species. *Nucleic Acids Res.* **41**, D676–D683 (2013).
5. Van Bel, M. *et al.* PLAZA 4.0: An integrative resource for functional, evolutionary and comparative plant genomics. *Nucleic Acids Res.* **46**, D1190–D1196 (2018).
6. Proost, S. *et al.* PLAZA: a comparative genomics resource to study gene and genome evolution in plants. *Plant Cell* **21**, 3718–3731 (2009).
7. Smith, D. R., Hua, J., Lee, R. W. & Keeling, P. J. Relative rates of evolution among the three genetic compartments of the red alga Porphyra differ from those of green plants and do not correlate with genome architecture. *Mol. Phylogenet. Evol.* **65**, 339–344 (2012).
8. Gray, M. W. *et al.* The draft nuclear genome sequence and predicted mitochondrial proteome of Andalucia godoyi, a protist with the most gene-rich and bacteria-like mitochondrial genome. *BMC Biol.* **18**, 22 (2020).
9. Shoguchi, E. *et al.* Draft assembly of the symbiodinium minutum nuclear genome reveals dinoflagellate gene structure. *Curr. Biol.* **23**, 1399–1408 (2013).
10. Keeling, P. J. *et al.* The Marine Microbial Eukaryote Transcriptome Sequencing Project (MMETSP): Illuminating the Functional Diversity of Eukaryotic Life in the Oceans through Transcriptome Sequencing. *PLoS Biol.* **12**, e1001889 (2014).
11. Muñoz-Gómez, S. A. *et al.* An updated phylogeny of the alphaproteobacteria reveals that the parasitic rickettsiales and holosporales have independent origins. *Elife* **8**, e42535 (2019).
12. Martijn, J., Vosseberg, J., Guy, L., Offre, P. & Ettema, T. J. G. Deep mitochondrial origin outside the sampled alphaproteobacteria. *Nature* **557**, 101–105 (2018).
13. Burki, F. The eukaryotic tree of life from a global phylogenomic perspective. *Cold Spring Harb. Perspect. Biol.* **6**, a016147 (2014).
14. Keeling, P. J. & Burki, F. Progress towards the Tree of Eukaryotes. *Curr. Biol.* **29**, R808–R817 (2019).
15. Betts, H. C. *et al.* Integrated genomic and fossil evidence illuminates life's early evolution and eukaryote origin. *Nat. Ecol. Evol.* **2**, 1556–1562 (2018).
16. Rodríguez-Ezpeleta, N. & Embley, T. M. The SAR11 group of alpha-proteobacteria is not related to the origin of mitochondria. *PLoS One* **7**, e30520 (2012).

17. Wang, Z. & Wu, M. An integrated phylogenomic approach toward pinpointing the origin of mitochondria. *Sci. Rep.* **5**, 7949 (2015).
18. Gao, F. *et al.* The All-Data-Based Evolutionary Hypothesis of Ciliated Protists with a Revised Classification of the Phylum Ciliophora (Eukaryota, Alveolata). *Sci. Rep.* **6**, 24874 (2016).
19. Dos Reis, M. *et al.* Uncertainty in the Timing of Origin of Animals and the Limits of Precision in Molecular Timescales. *Curr. Biol.* **25**, 2939–2950 (2015).
20. Foster, C. S. P. & Ho, S. Y. W. Strategies for partitioning clockmodels in phylogenomic dating: Application to the angiosperm evolutionary timescale. *Genome Biol. Evol.* **9**, 2752–2763 (2017).
21. Dos Reis, M. *et al.* Using phylogenomic data to explore the effects of relaxed clocks and calibration strategies on divergence time estimation: Primates as a test case. *Syst. Biol.* **67**, 594–615 (2018).
22. McGowen, M. R. *et al.* Phylogenomic Resolution of the Cetacean Tree of Life Using Target Sequence Capture. *Syst. Biol.* **69**, 479–501 (2020).
23. Fan, L. *et al.* Phylogenetic analyses with systematic taxon sampling show that mitochondria branch within Alphaproteobacteria. *Nat. Ecol. Evol.* **4**, 1213–1219 (2020).
24. Brown, R. P. & Yang, Z. Rate variation and estimation of divergence times using strict and relaxed clocks. *BMC Evol. Biol.* **11**, 271 (2011).
25. Le, S. Q. & Gascuel, O. An improved general amino acid replacement matrix. *Mol. Biol. Evol.* **25**, 1307–1320 (2008).
26. Tavaré, S. Some probabilistic and statistical problems in the analysis of DNA sequences. *American Mathematical Society: Lectures on Mathematics in the Life Sciences* vol. 17 57–86 (1986).
27. Wang, S., Meade, A., Lam, H.-M. & Luo, H. Evolutionary Timeline and Genomic Plasticity Underlying the Lifestyle Diversity in Rhizobiales. *mSystems* **5**, e00438-20 (2020).
28. Soo, R. M., Hemp, J., Parks, D. H., Fischer, W. W. & Hugenholtz, P. On the origins of oxygenic photosynthesis and aerobic respiration in Cyanobacteria. *Science (80-.).* **355**, 1436–1440 (2017).
29. Battistuzzi, F. U. & Hedges, S. B. A major clade of prokaryotes with ancient adaptations to life on land. *Mol. Biol. Evol.* **26**, 335–343 (2009).
30. Schwabe, I. BayesTwin: An R Package for Bayesian Inference of Item-Level Twin Data. *J. Open Res. Softw.* **5**, 33 (2017).
31. Rannala, B. & Yang, Z. Inferring speciation tunes under an episodic molecular clock. *Syst. Biol.* **56**, 453–466 (2007).
32. Yang, Z. & Rannala, B. Bayesian estimation of species divergence times under a molecular clock using multiple fossil calibrations with soft bounds. *Mol. Biol. Evol.* (2006) doi:10.1093/molbev/msj024.
33. Lartillot, N., Lepage, T. & Blanquart, S. PhyloBayes 3: A Bayesian software package for phylogenetic reconstruction and molecular dating. *Bioinformatics* **25**, 2286–2288 (2009).

34. Lartillot, N., Blanquart, S. & Lepage, T. PhyloBayes V4.1 Manual. (2015).
35. Bouckaert, R. *et al.* BEAST 2.5: An advanced software platform for Bayesian evolutionary analysis. *PLoS Comput. Biol.* **15**, e1006650 (2019).
36. Roger, A. J., Muñoz-Gómez, S. A. & Kamikawa, R. The Origin and Diversification of Mitochondria. *Current Biology* vol. 27 R1177–R1192 (2017).
37. Yang, D., Oyaizu, Y., Oyaizu, H., Olsen, G. J. & Woese, C. R. Mitochondrial origins. *Proc. Natl. Acad. Sci. U. S. A.* **82**, 4443–4447 (1985).
38. Andersson, S. G. E. *et al.* The genome sequence of Rickettsia prowazekii and the origin of mitochondria. *Nature* **396**, 133–140 (1998).
39. Georgiades, K., Madoui, M. A., Le, P., Robert, C. & Raoult, D. Phylogenomic analysis of odyssella thessalonicensis fortifies the common origin of rickettsiales, pelagibacter ubique and reclimonas americana mitochondrion. *PLoS One* **6**, e24857 (2011).
40. Thrash, J. C. *et al.* Phylogenomic evidence for a common ancestor of mitochondria and the SAR11 clade. *Sci. Rep.* **1**, 13 (2011).
41. Luo, H. Evolutionary origin of a streamlined marine bacterioplankton lineage. *ISME J.* **9**, 1423–1433 (2015).
42. Kishino, H., Miyata, T. & Hasegawa, M. Maximum likelihood inference of protein phylogeny and the origin of chloroplasts. *J. Mol. Evol.* **31**, 151–160 (1990).
43. Kishino, H. & Hasegawa, M. Evaluation of the maximum likelihood estimate of the evolutionary tree topologies from DNA sequence data, and the branching order in hominoidea. *J. Mol. Evol.* **29**, 170–179 (1989).
44. Shimodaira, H. & Hasegawa, M. Multiple comparisons of log-likelihoods with applications to phylogenetic inference. *Molecular Biology and Evolution* vol. 16 1114–1116 (1999).
45. Strimmer, K. & Rambaut, A. Inferring confidence sets of possibly misspecified gene trees. *Proc. R. Soc. B Biol. Sci.* **269**, 137–142 (2002).
46. Shimodaira, H. An approximately unbiased test of phylogenetic tree selection. *Syst. Biol.* **51**, 492–508 (2002).
47. Rodriguez-R, L. M. *et al.* How much do rRNA gene surveys underestimate extant bacterial diversity? *Appl. Environ. Microbiol.* **84**, e00014-18 (2018).
48. Meade, A. & Pagel, M. BayesTraits V3 Manual. (2016).
49. Letunic, I. & Bork, P. Interactive Tree Of Life (iTOL) v4: recent updates and new developments. *Nucleic Acids Res.* **47**, W256–W259 (2019).
50. Kumar, S., Stecher, G. & Tamura, K. MEGA7: Molecular Evolutionary Genetics Analysis Version 7.0 for Bigger Datasets. *Mol. Biol. Evol.* **33**, 1870–1874 (2016).
51. Stöver, B. C. & Müller, K. F. TreeGraph 2: Combining and visualizing evidence from different phylogenetic analyses. *BMC Bioinformatics* (2010) doi:10.1186/1471-2105-11-7.
52. Yu, G., Smith, D. K., Zhu, H., Guan, Y. & Lam, T. T. Y. Ggtree: an R Package for Visualization and Annotation of Phylogenetic Trees With Their Covariates

and Other Associated Data. *Methods Ecol. Evol.* **8**, 28–36 (2017).
53. Hall, T. A. BIOEDIT: a user-friendly biological sequence alignment editor and analysis program for Windows 95/98/ NT. *Nucleic Acids Symp. Ser.* **41**, 95–98 (1999).
54. Puttick, M. N. MCMCtreeR: Functions to prepare MCMCtree analyses and visualize posterior ages on trees. *Bioinformatics* **35**, 5321–5322 (2019).
55. Knoll, A. H. The Multiple Origins of Complex Multicellularity. *Annu. Rev. Earth Planet. Sci.* **39**, 217–239 (2011).
56. Turner, E. C. & Kamber, B. S. Arctic Bay Formation, Borden Basin, Nunavut (Canada): Basin evolution, black shale, and dissolved metal systematics in the Mesoproterozoic ocean. *Precambrian Res.* **208**–**211**, 1–18 (2012).
57. Ogg, J. G. Triassic. in *The Geologic Time Scale 2012* vols 1–2 681–730 (2012).
58. Clarke, J. T., Warnock, R. C. M. & Donoghue, P. C. J. Establishing a time-scale for plant evolution. *New Phytol.* **192**, 266–301 (2011).
59. Morris, J. L. *et al.* The timescale of early land plant evolution. *Proc. Natl. Acad. Sci. U. S. A.* **115**, E2274–E2283 (2018).
60. Baldwin, C. T., Strother, P. K., Beck, J. H. & Rose, E. Palaeoecology of the Bright Angel Shale in the eastern Grand Canyon, Arizona, USA, incorporating sedimentological, ichnological and palynological data. *Geol. Soc. Spec. Publ.* **228**, 213–236 (2004).
61. Clark, J. W. & Donoghue, P. C. J. Constraining the timing of whole genome duplication in plant evolutionary history. *Proc. R. Soc. B Biol. Sci.* **284**, 20170912 (2017).
62. Xiao, S., Knoll, A. H., Yuan, X. & Pueschel, C. M. Phosphatized multicellular algae in the Neoproterozoic Doushantuo Formation, China, and the early evolution of florideophyte red algae. *Am. J. Bot.* **91**, 214–227 (2004).
63. Knoll, A. H. & Xiao, S. hai. On the age of the Doushantuo Formation. *Acta Micropalaeontologica Sin.* **16**, 225–236 (1999).
64. Parfrey, L. W., Lahr, D. J. G., Knoll, A. H. & Katz, L. A. Estimating the timing of early eukaryotic diversification with multigene molecular clocks. *Proc. Natl. Acad. Sci. U. S. A.* **108**, 13624–13629 (2011).
65. Zhongying, Z. Clastic facies microfossils from the Chuanlinggou Formation (1800 Ma) near Jixian, North China. *J. Micropalaeontology* **5**, 9–16 (1986).
66. Peng, Y., Bao, H. & Yuan, X. New morphological observations for Paleoproterozoic acritarchs from the Chuanlinggou Formation, North China. *Precambrian Res.* **168**, 223–232 (2009).
67. Lamb, D. M., Awramik, S. M., Chapman, D. J. & Zhu, S. Evidence for eukaryotic diversification in the ∼1800 million-year-old Changzhougou Formation, North China. *Precambrian Res.* **173**, 93–104 (2009).
68. Lu, S., Yang, C., Zhu, S. & Mei, H. The Precambrian continental crust from sastern Hebei to Jixian. in *Tianjin 30th International Geological Congress, Beijing: Geological Publishing House* (1996).
69. Yang, E. C. *et al.* Divergence time estimates and the evolution of major lineages in the florideophyte red algae. *Sci. Rep.* **6**, 21361 (2016).

70. McIlroy, D., Green, O. R. & Brasier, M. D. Palaeobiology and evolution of the earliest agglutinated Foraminifera: Platysolenites, Spirosolenites and related forms. *Lethaia* **34**, 13–29 (2001).
71. Gradstein, F. M., Ogg, J. G., Schmitz, M. D. & Ogg, G. M. *The geologic time scale*. *The Geologic Time Scale 2012* (2012). doi:10.1016/C2011-1-08249-8.
72. Benton, M. J. *et al.* Constraints on the timescale of animal evolutionary history. *Palaeontologia Electronica* vol. 18 (2015).
73. Shu, D. G. *et al.* Lower Cambrian vertebrates from south China. *Nature* **402**, 42–46 (1999).
74. Hou, X.-G. *et al. The Cambrian fossils of Chengjiang, China: The flowering of early animal life*. *The Cambrian Fossils of Chengjiang, China: The Flowering of Early Animal Life* (2008). doi:10.1002/9780470999950.
75. Yuan, X., Chen, Z., Xiao, S., Zhou, C. & Hua, H. An early Ediacaran assemblage of macroscopic and morphologically differentiated eukaryotes. *Nature* **470**, 390–393 (2011).
76. Blair, J. E. & Hedges, S. B. Molecular phylogeny and divergence times of deuterostome animals. *Mol. Biol. Evol.* **22**, 2275–2284 (2005).
77. Fedonkin, M. A., Simonetta, A. & Ivantsov, A. Y. New data on Kimberella, the Vendian mollusc-like organism (White Sea region, Russia): Palaeoecological and evolutionary implications. in *Geological Society Special Publication* (2007). doi:10.1144/SP286.12.
78. Butterfield, N. J., KNOLL, A. H. & SWETT, K. Paleobiology of the Neoproterozoic Svanbergfjellet Formation, Spitsbergen. *Lethaia* **27**, 76–76 (1994).
79. Schopf, J. W. Microflora of the Bitter Springs Formation, Late Precambrian, Central Australia. *J. Paleontol.* **42**, 651–688 (1968).
80. Feuda, R. *et al.* Improved Modeling of Compositional Heterogeneity Supports Sponges as Sister to All Other Animals. *Curr. Biol.* **27**, 3864–3870.e4 (2017).
81. Pisani, D. *et al.* Genomic data do not support comb jellies as the sister group to all other animals. *Proc. Natl. Acad. Sci. U. S. A.* **112**, 15402–15407 (2015).
82. Nettersheim, B. J. *et al.* Putative sponge biomarkers in unicellular Rhizaria question an early rise of animals. *Nat. Ecol. Evol.* **3**, 577–581 (2019).
83. Cartwright, P. & Collins, A. Fossils and phylogenies: Integrating multiple lines of evidence to investigate the origin of early major metazoan lineages. *Integr. Comp. Biol.* **47**, 744–751 (2007).
84. Hedges, S. B., Blair, J. E., Venturi, M. L. & Shoe, J. L. A molecular timescale of eukaryote evolution and the rise of complex multicellular life. *BMC Evol. Biol.* **4**, 2 (2004).
85. Loron, C. C. *et al.* Early fungi from the Proterozoic era in Arctic Canada. *Nature* vol. 570 232–235 (2019).
86. Bengtson, S. *et al.* Fungus-like mycelial fossils in 2.4-billion-year-old vesicular basalt. *Nat. Ecol. Evol.* **1**, (2017).
87. Berbee, M. L., James, T. Y. & Strullu-Derrien, C. Early Diverging Fungi: Diversity and Impact at the Dawn of Terrestrial Life. *Annu. Rev. Microbiol.* **71**,

41–60 (2017).
88. Mark, D. F. *et al.* 40Ar/39Ar dating of hydrothermal activity, biota and gold mineralization in the Rhynie hot-spring system, Aberdeenshire, Scotland. *Geochim. Cosmochim. Acta* **75**, 555–569 (2011).
89. Schoene, B., Latkoczy, C., Schaltegger, U. & Günther, D. A new method integrating high-precision U-Pb geochronology with zircon trace element analysis (U-Pb TIMS-TEA). *Geochim. Cosmochim. Acta* **74**, 7144–7159 (2010).
90. Zhang, Y. & Golubic, S. Endolithic microfossils (cyanophyta) from early Proterozoic stromatolites, Hebei, China. *Acta Micropalaeontologica Sin.* **4**, 1–3 (1987).
91. Sánchez-Baracaldo, P. Origin of marine planktonic cyanobacteria. *Sci. Rep.* **5**, 17418 (2015).
92. Sánchez-Baracaldo, P., Raven, J. A., Pisani, D. & Knoll, A. H. Early photosynthetic eukaryotes inhabited low-salinity habitats. *Proc. Natl. Acad. Sci.* (2017) doi:10.1073/pnas.1620089114.
93. Marshall, C. R. Using the Fossil Record to Evaluate Timetree Timescales. *Frontiers in Genetics* vol. 10 (2019).
94. Zhang, H., Sun, Y., Zeng, Q., Crowe, S. A. & Luo, H. Snowball Earths, population bottlenecks, and the evolution of marine photosynthetic bacteria. *bioRxiv* (2021) doi:10.1101/2020.11.24.395392.
95. Wolfe, J. M. & Fournier, G. P. Horizontal gene transfer constrains the timing of methanogen evolution. *Nat. Ecol. Evol.* **2**, 897–903 (2018).
96. de Tezanos Pinto, P., Kust, A., Devercelli, M. & Kozlíková-Zapomělová, E. Morphological traits in nitrogen fixing heterocytous cyanobacteria: possible links between morphology and eco-physiology. *Hydrobiologia* **764**, 271–281 (2016).
97. Tomitani, A., Knoll, A. H., Cavanaugh, C. M. & Ohno, T. The evolutionary diversification of cyanobacteria: Molecular-phylogenetic and paleontological perspectives. *Proc. Natl. Acad. Sci. U. S. A.* **103**, 5442–5447 (2006).
98. Amard, B. & Bertrand-Sarfati, J. Microfossils in 2000 Ma old cherty stromatolites of the Franceville Group, Gabon. *Precambrian Res.* **81**, 197–221 (1997).
99. Horodyski, R. J. & Allan Donaldson, J. Microfossils from the Middle Proterozoic Dismal Lakes Groups, Arctic Canada. *Precambrian Res.* **11**, 125–159 (1980).
100. Sánchez-Baracaldo, P., Ridgwell, A. & Raven, J. A. A neoproterozoic transition in the marine nitrogen cycle. *Curr. Biol.* **24**, 652–657 (2014).
101. Kump, L. R. The rise of atmospheric oxygen. *Nature* **451**, 277–278 (2008).
102. Schirrmeister, B. E., De Vos, J. M., Antonelli, A. & Bagheri, H. C. Evolution of multicellularity coincided with increased diversification of cyanobacteria and the Great Oxidation Event. *Proc. Natl. Acad. Sci. U. S. A.* **110**, 1791–1796 (2013).
103. Kopp, R. E., Kirschvink, J. L., Hilburn, I. A. & Nash, C. Z. The

paleoproterozoic snowball Earth: A climate disaster triggered by the evolution of oxygenic photosynthesis. *Proceedings of the National Academy of Sciences of the United States of America* vol. 102 11131–11136 (2005).
104. Crowe, S. A. *et al.* Atmospheric oxygenation three billion years ago. *Nature* **501**, 535–538 (2013).
105. Planavsky, N. J. *et al.* Evidence for oxygenic photosynthesis half a billion years before the Great Oxidation Event. *Nat. Geosci.* **7**, 283–286 (2014).
106. Javaux, E. J. Challenges in evidencing the earliest traces of life. *Nature* vol. 572 451–460 (2019).
107. Strauss, H. The sulfur isotopic record of Precambrian sulfates: new data and a critical evaluation of the existing record. *Precambrian Res.* **63**, 225–246 (1993).
108. Van Kranendonk, M. J., Philippot, P., Lepot, K., Bodorkos, S. & Pirajno, F. Geological setting of Earth's oldest fossils in the ca. 3.5 Ga Dresser Formation, Pilbara Craton, Western Australia. *Precambrian Res.* **167**, 93–124 (2008).
109. Allwood, A. C., Walter, M. R., Burch, I. W. & Kamber, B. S. 3.43 billion-year-old stromatolite reef from the Pilbara Craton of Western Australia: Ecosystem-scale insights to early life on Earth. *Precambrian Res.* **158**, 198–227 (2007).
110. Allègre, C. J., Manhès, G. & Göpel, C. The age of the Earth. *Geochim. Cosmochim. Acta* **59**, 1445–1456 (1995).
111. Schirrmeister, B. E., Sanchez-Baracaldo, P. & Wacey, D. Cyanobacterial evolution during the Precambrian. *Int. J. Astrobiol.* **15**, 187–204 (2016).
112. de Vries, J. & Archibald, J. M. Plastid genomes. *Current Biology* vol. 28 R336–R337 (2018).
113. Battistuzzi, F. U., Feijao, A. & Hedges, S. B. A genomic timescale of prokaryote evolution: Insights into the origin of methanogenesis, phototrophy, and the colonization of land. *BMC Evol. Biol.* **4**, 44 (2004).
114. Luo, H., Csuros, M., Hughes, A. L. & Moran, M. A. Evolution of divergent life history strategies in marine alphaproteobacteria. *MBio* **4**, e00373-13 (2013).
115. Weinert, L. A., Werren, J. H., Aebi, A., Stone, G. N. & Jiggins, F. M. Evolution and diversity of Rickettsia bacteria. *BMC Biol.* **7**, 6 (2009).
116. Chriki-Adeeb, R. & Chriki, A. Estimating divergence times and substitution rates in Rhizobia. *Evol. Bioinforma.* **12**, 87–97 (2016).
117. Young, J. P. W. & Johnston, A. W. B. The evolution of specificity in the legume-rhizobium symbiosis. *Trends Ecol. Evol.* **4**, 341–349 (1989).
118. Mutch, L. A. & Young, J. P. W. Diversity and specificity of Rhizobium leguminosarum biovar viciae on wild and cultivated legumes. *Mol. Ecol.* **13**, 2435–2444 (2004).
119. Wang, D., Yang, S., Tang, F. & Zhu, H. Symbiosis specificity in the legume - rhizobial mutualism. *Cellular Microbiology* vol. 14 334–342 (2012).
120. Masson-Boivin, C., Giraud, E., Perret, X. & Batut, J. Establishing nitrogen-fixing symbiosis with legumes: how many rhizobium recipes? *Trends in Microbiology* vol. 17 458–466 (2009).
121. Ochman, H. & Wilson, A. C. Evolution in bacteria: Evidence for a universal

substitution rate in cellular genomes. *J. Mol. Evol.* **26**, 74–86 (1987).
122. Garrido-Oter, R. *et al.* Modular Traits of the Rhizobiales Root Microbiota and Their Evolutionary Relationship with Symbiotic Rhizobia. *Cell Host Microbe* **24**, 155–167.e5 (2018).
123. Yeoh, Y. K. *et al.* Evolutionary conservation of a core root microbiome across plant phyla along a tropical soil chronosequence. *Nat. Commun.* **8**, 215 (2017).
124. Moran, N. A., Munson, M. A., Baumann, P. & Ishikawa, H. A molecular clock in endosymbiotic bacteria is calibrated using the insect hosts. *Proc. R. Soc. B Biol. Sci.* **253**, 167–171 (1993).
125. Thomas, S., Alexander, W., Gilligan, J. & Rikihisa, Y. The importance of Rickettsiales infections. in *Rickettsiales: Biology, Molecular Biology, Epidemiology, and Vaccine Development* 3–21 (2016). doi:10.1007/978-3-319-46859-4_1.
126. Werren, J. H., Baldo, L. & Clark, M. E. Wolbachia: Master manipulators of invertebrate biology. *Nature Reviews Microbiology* vol. 6 741–751 (2008).
127. Bordenstein, S. R. Symbiosis and the origin of species. in *Insect Symbiosis* 283–304 (2003). doi:10.1201/9780203009918.
128. Perlman, S. J., Hunter, M. S. & Zchori-Fein, E. The emerging diversity of Rickettsia. *Proceedings of the Royal Society B: Biological Sciences* vol. 273 2097–2106 (2006).
129. Klinges, J. G. *et al.* Phylogenetic, genomic, and biogeographic characterization of a novel and ubiquitous marine invertebrate-associated Rickettsiales parasite, Candidatus Aquarickettsia rohweri, gen. nov., sp. nov. *ISME J.* **13**, 2938–2953 (2019).
130. Kawafune, K., Hongoh, Y., Hamaji, T. & Nozaki, H. Molecular identification of rickettsial endosymbionts in the non-phagotrophic volvocalean green algae. *PLoS One* **7**, e31749 (2012).
131. Kawafune, K., Hongoh, Y. & Nozaki, H. A rickettsial endosymbiont inhabiting the cytoplasm of Volvox carteri (Volvocales, Chlorophyceae). *Phycologia* **53**, 95–99 (2014).
132. Montagna, M. *et al.* ‘Candidatus Midichloriaceae’ fam. Nov. (Rickettsiales), an ecologically: Widespread clade of intracellular alphaproteobacteria. *Appl. Environ. Microbiol.* **79**, 3241–3248 (2013).
133. Vannini, C., Petroni, G., Verni, F. & Rosati, G. A bacterium belonging to the Rickettsiaceae family inhabits the cytoplasm of the marine ciliate Diophrys appendiculata (Ciliophora, Hypotrichia). *Microb. Ecol.* **49**, 434–442 (2005).
134. Szokoli, F. *et al.* Disentangling the taxonomy of Rickettsiales and description of two novel symbionts (‘Candidatus Bealeia paramacronuclearis’ and ‘Candidatus Fokinia cryptica’) sharing the cytoplasm of the ciliate protist Paramecium biaurelia. *Appl. Environ. Microbiol.* **82**, 7236–7247 (2016).
135. Schrallhammer, M. *et al.* ‘Candidatus Megaira polyxenophila’ gen. nov., sp. nov.: Considerations on Evolutionary History, Host Range and Shift of Early Divergent Rickettsiae. *PLoS One* **8**, e72581 (2013).
136. Merhej, V. & Raoult, D. Rickettsial evolution in the light of comparative

genomics. *Biological Reviews* vol. 86 379–405 (2011).
137. El Karkouri, K., Pontarotti, P., Raoult, D. & Fournier, P. E. Origin and evolution of rickettsial plasmids. *PLoS One* **11**, (2016).
138. De La Fuente, J., Estrada-Peña, A., Cabezas-Cruz, A. & Brey, R. Flying ticks: Anciently evolved associations that constitute a risk of infectious disease spread. *Parasites and Vectors* vol. 8 538 (2015).
139. Krawczak, F. S., Labruna, M. B., Hecht, J. A., Paddock, C. D. & Karpathy, S. E. Genotypic Characterization of Rickettsia bellii Reveals Distinct Lineages in the United States and South America. *Biomed Res. Int.* **2018**, (2018).
140. Baldridge, G. D. *et al.* Wide dispersal and possible multiple origins of low-copy-number plasmids in Rickettsia species associated with blood-feeding arthropods. *Appl. Environ. Microbiol.* **76**, 1718–1731 (2010).
141. Tomassone, L., Portillo, A., Nováková, M., De Sousa, R. & Oteo, J. A. Neglected aspects of tick-borne rickettsioses. *Parasites and Vectors* vol. 11 (2018).
142. Sachman-Ruiz, B. & Quiroz-Castañeda, R. E. Genomics of Rickettsiaceae: An Update. in *Farm Animals Diseases, Recent Omic Trends and New Strategies of Treatment* (2018). doi:10.5772/intechopen.74563.
143. Castelli, M. *et al.* Deianiraea, an extracellular bacterium associated with the ciliate Paramecium, suggests an alternative scenario for the evolution of Rickettsiales. *ISME J.* **13**, 2280–2294 (2019).
144. Walker, D. H. Chapter 38 Rickettsiae. in *Medical Microbiology* (ed. Baron, S.) (The University of Texas Medical Branch at Galveston, 1996).
145. Kang, Y. J. *et al.* Extensive diversity of Rickettsiales bacteria in two species of ticks from China and the evolution of the Rickettsiales. *BMC Evol. Biol.* (2014) doi:10.1186/s12862-014-0167-2.
146. Modeo, L. *et al.* Candidatus Trichorickettsia mobilis'', a Rickettsiales bacterium, can be transiently transferred from the unicellular eukaryote Paramecium to the planarian Dugesia japonica. *PeerJ* **2020**, e8977 (2020).
147. Dos Reis, M., Donoghue, P. C. J. & Yang, Z. Bayesian molecular clock dating of species divergences in the genomics era. *Nature Reviews Genetics* (2016) doi:10.1038/nrg.2015.8.
148. Fitzpatrick, D. A., Creevey, C. J. & McInerney, J. O. Genome phylogenies indicate a meaningful α-proteobacterial phylogeny and support a grouping of the mitochondria with the Rickettsiales. *Mol. Biol. Evol.* **23**, 74–85 (2006).
149. Ho, S. Y. W. & Duchêne, S. Molecular-clock methods for estimating evolutionary rates and timescales. *Mol. Ecol.* **23**, 5947–5965 (2014).
150. Törnqvist, L., Vartia, P. & Vartia, Y. O. How should relative changes be measured? *Am. Stat.* **39**, 43–46 (1985).
151. Jeffreys, H. *Theory of Probability*. (Clarendon Press, Oxford, 1961).
