# Supplementary material for: Dating Alphaproteobacteria evolution with eukaryotic fossils
Source: Nat Commun. 2021 Jun 3;12:3324. doi: 10.1038/s41467-021-23645-4 (PMC8175736; doi:10.1038/s41467-021-23645-4)
Supplement: Supplementary file 3 — Description of Additional Supplementary Files [file 41467_2021_23645_MOESM3_ESM.pdf]

Description of Additional Supplementary Files

File Name: Supplementary Data 1
Description: Genes used in MCMCTree analysis.

File Name: Supplementary Data 2
Description: Different dating schemes used in MCMCTree analysis. The relationships between the node ids and their phylogenetic placement are illustrated in Supplementary Fig. 2.

File Name: Supplementary Data 3
Description: Genes and species used in mcmc3r.
