# Supplementary material for: Dating Alphaproteobacteria evolution with eukaryotic fossils
Source: Nat Commun. 2021 Jun 3;12:3324. doi: 10.1038/s41467-021-23645-4 (PMC8175736; doi:10.1038/s41467-021-23645-4)
Supplement: Supplementary file 5 — Reporting Summary [file 41467_2021_23645_MOESM5_ESM.pdf]

nature research

Corresponding author(s): Haiwei Luo

Last updated by author(s): May 6, 2021

Reporting Summary

Nature Research wishes to improve the reproducibility of the work that we publish. This form provides structure for consistency and transparency in reporting. For further information on Nature Research policies, see our Editorial Policies and the Editorial Policy Checklist.

Statistics

For all statistical analyses, confirm that the following items are present in the figure legend, table legend, main text, or Methods section.

n/a	Confirmed	
☐	☒	The exact sample size (*n*) for each experimental group/condition, given as a discrete number and unit of measurement
☐	☒	A statement on whether measurements were taken from distinct samples or whether the same sample was measured repeatedly
☐	☒	The statistical test(s) used AND whether they are one- or two-sided<br>*Only common tests should be described solely by name; describe more complex techniques in the Methods section.*
☒	☐	A description of all covariates tested
☒	☐	A description of any assumptions or corrections, such as tests of normality and adjustment for multiple comparisons
☐	☒	A full description of the statistical parameters including central tendency (e.g. means) or other basic estimates (e.g. regression coefficient) AND variation (e.g. standard deviation) or associated estimates of uncertainty (e.g. confidence intervals)
☐	☒	For null hypothesis testing, the test statistic (e.g. *F*, *t*, *r*) with confidence intervals, effect sizes, degrees of freedom and *P* value noted<br>*Give P values as exact values whenever suitable.*
☐	☒	For Bayesian analysis, information on the choice of priors and Markov chain Monte Carlo settings
☒	☐	For hierarchical and complex designs, identification of the appropriate level for tests and full reporting of outcomes
☒	☐	Estimates of effect sizes (e.g. Cohen's *d*, Pearson's *r*), indicating how they were calculated

Our web collection on statistics for biologists contains articles on many of the points above.

Software and code

Policy information about availability of computer code

Data collection	No software was used for data collection.
Data analysis	Sequence alignment: MAFFT v7.222<br>Alignment trimming: TrimAl v1.4<br>Phylogenetic reconstruction: IQ-Tree v1.6.11<br>Estimation of divergence time: MCMCTree v4.9j, PhyloBayes v4.1b, BEAST v2.6.3<br>Selection of the clock model: mcmc3r<br>Composite chronogram: BayesTwin<br>MCMC Diagnostics: Tracer v1.6<br>Ancestral trait reconstruction: BayesTraits v3.0.2<br>Data visualization: MCMCTreeR, FigTree v1.4.3, iTOL v4, MEGA v7, TreeGraph v2.5, ggtree v2.0.4, BioEdit v7.0.5.3<br>Sequence processing: BioRuby v2.1<br>Custom scripts generating the results are available at https://doi.org/10.6084/m9.figshare.12763547.

For manuscripts utilizing custom algorithms or software that are central to the research but not yet described in published literature, software must be made available to editors and reviewers. We strongly encourage code deposition in a community repository (e.g. GitHub). See the Nature Research guidelines for submitting code & software for further information.

Data

Policy information about availability of data

All manuscripts must include a data availability statement. This statement should provide the following information, where applicable:

- Accession codes, unique identifiers, or web links for publicly available datasets
- A list of figures that have associated raw data
- A description of any restrictions on data availability

All of the data are present in the paper or available at the online repository https://doi.org/10.6084/m9.figshare.12763547. In particular, the mito- and nuclear-encoded datasets can be found in the repository under the folders dating-aa/mito-encoded and dating-aa/nuclear-encoded, respectively. The genome sources are listed in Supplementary Table 1.

Field-specific reporting

Please select the one below that is the best fit for your research. If you are not sure, read the appropriate sections before making your selection.

- [x] Life sciences
- [ ] Behavioural & social sciences
- [ ] Ecological, evolutionary & environmental sciences

For a reference copy of the document with all sections, see nature.com/documents/nr-reporting-summary-flat.pdf

Life sciences study design

All studies must disclose on these points even when the disclosure is negative.

Sample size	The effective size of the vast majority of the parameters in MCMCTree analysis was above 200.
Data exclusions	No data were excluded from analyses.
Replication	MCMC molecular clock dating analyses were replicated for twice to ensure that convergence has reached.
Randomization	Not relevant. Our study is a bioinformatic study, which did not involve any animals/participants/plants/microbes.
Blinding	Not relevant. Our study is a bioinformatic study, which did not involve any animals/participants/plants/microbes.

Reporting for specific materials, systems and methods

We require information from authors about some types of materials, experimental systems and methods used in many studies. Here, indicate whether each material, system or method listed is relevant to your study. If you are not sure if a list item applies to your research, read the appropriate section before selecting a response.

Materials & experimental systems

n/a	Involved in the study	
x		Antibodies
x		Eukaryotic cell lines
x		Palaeontology and archaeology
x		Animals and other organisms
x		Human research participants
x		Clinical data
x		Dual use research of concern

Methods

n/a	Involved in the study	
x		ChIP-seq
x		Flow cytometry
x		MRI-based neuroimaging
